# Integrative multiomics evaluation reveals the importance of pseudouridine synthases in hepatocellular carcinoma

**DOI:** 10.3389/fgene.2022.944681

**Published:** 2022-11-10

**Authors:** Zhipeng Jin, Mengying Song, Jianping Wang, Wenjing Zhu, Dongxu Sun, Huayuan Liu, Guangjun Shi

**Affiliations:** ^1^ Graduate School of Dalian Medical University, Dalian, China; Department of Hepatobiliary Surgery, Qingdao Municipal Hospital, Qingdao, China; ^2^ Department of Operation Room, Cancer Hospital of Dalian University of Technology, Liaoning Cancer Hospital and Institute, Shenyang, China; ^3^ Clinical Research Center, Qingdao Municipal Hospital, Qingdao, China; ^4^ Department of Hepatobiliary Surgery, Qingdao Municipal Hospital, Qingdao, China

**Keywords:** pseudouridine synthase, HCC, biomarker, tumor microenvironment, multiomics data

## Abstract

**Background:** The pseudouridine synthases (PUSs) have been reported to be associated with cancers. However, their involvement in hepatocellular carcinoma (HCC) has not been well documented. Here, we assess the roles of PUSs in HCC.

**Methods:** RNA sequencing data of TCGA-LIHC and LIRI-JP were downloaded from the Cancer Genome Atlas (TCGA) and the International Cancer Genome Consortium (ICGC), respectively. GSE36376 gene expression microarray was downloaded from the Gene Expression Omnibus (GEO). Proteomics data for an HBV-related HCC cohort was obtained from the CPTAC Data Portal. The RT-qPCR assay was performed to measure the relative mRNA expression of genes in clinical tissues and cell lines. Diagnostic efficiency was evaluated by the ROC curve. Prognostic value was assessed using the Kaplan-Meier curve, Cox regression model, and time-dependent ROC curve. Copy number variation (CNV) was analyzed using the GSCA database. Functional analysis was carried out with GSEA, GSVA, and clusterProfiler package. The tumor microenvironment (TME) related analysis was performed using ssGSEA and the ESTIMATE algorithm.

**Results:** We identified 7 *PUSs* that were significantly upregulated in HCC, and 5 of them (*DKC1*, *PUS1*, *PUS7*, *PUSL1*, and *RPUSD3*) were independent risk factors for patients’ OS. Meanwhile, the protein expression of DKC1, PUS1, and PUS7 was also upregulated and related to poor survival. Both mRNA and protein of these PUSs were highly diagnostic of HCC. Moreover, the CNV of *PUS1*, *PUS7*, *PUS7L*, and *RPUSD2* was also associated with prognosis. Further functional analysis revealed that PUSs were mainly involved in pathways such as genetic information processing, substance metabolism, cell cycle, and immune regulation.

**Conclusion:** PUSs may play crucial roles in HCC and could be used as potential biomarkers for the diagnosis and prognosis of patients.

## Introduction

Hepatocellular carcinoma remains a global health challenge as one of the most malignant malignancies (Llovet et al., 2021). Early diagnosis and clinical intervention, together with advances in surgical methods and the development of anti-tumor therapies, have led to significant advances in the treatment of HCC. However, median survival time for patients with advanced HCC is only 2–3 years (Yang et al., 2019). Even in early HCC cases suitable for surgery, the 5-year recurrence rate after hepatectomy is close to 70% (Roayaie et al., 2013). The high heterogeneity of HCC is a massive challenge to improving clinical outcomes. Hence, there is an urgent need to explore the complex functional pathways and molecular mechanisms behind HCC, to develop new biomarkers for early diagnosis, prognosis and relapse prediction, and to identify new therapeutic targets (Llovet et al., 2018).

Post-transcriptional modifications could affect RNA stability, localization, structure, splicing, or function (Roundtree et al., 2017). And its deregulation has been linked to human diseases, including tumorigenesis (Jonkhout et al., 2017). RNA modifications regulate cancer cell fate by modulating cell survival, differentiation, migration and drug resistance (Delaunay and Frye, 2019). Pseudouridine (5-ribosyluracil, ψ) is the 5-ribosyl isomer of uridine, and is the most abundant type of RNA modification. It is also known as the ‘fifth nucleotide’ (Charette and Gray, 2000). Enzymes responsible for this modification are pseudouridine synthase (PUS). A total of thirteen PUSs have been identified in humans and are classified into two categories, RNA-dependent and RNA-independent PUSs (Supplementary Table S1). Of these, Dyskerin, the only RNA-dependent PUS, is a nucleolar protein encoded by the Dyskerin Pseudouridine Synthase 1 (*DKC1*) gene. It is present in small nucleolar ribonucleoprotein particles and is responsible for converting specific uridine residues of ribosomal (r) RNA into pseudouridine. And twelve RNA-independent PUSs can modify target RNAs by directly recognising sequences or RNA structures (Li et al., 2016).

Pseudouridine in cancer cells has emerged as a therapeutic target for cancer (Nombela et al., 2021). A recent study found that *DKC1* was a potential therapeutic target in colorectal cancer, and its increased expression was associated with poor prognosis (Kan et al., 2021). PUS7 was a targetable regulator of glioblastoma growth (Zhang et al., 2021a), and its increased expression was associated with reduced patient survival. The application of PUS7 inhibitors inhibited tumorigenesis and prolonged the life span of tumor-bearing mice (Cui et al., 2021). In ovarian cancer, both mRNA and protein expression of PUS7 were higher in cancer tissues than in normal tissues (Li et al., 2021). All these emerging findings showed the potential of PUSs as cancer biomarkers. Therefore more research is necessary to explore the importance of PUSs in cancer.

Currently, there is a lack of research on PUSs in HCC. To address this scientific gap, we integrated transcriptomic, genomic and proteomic data to assess the diagnostic, prognostic and therapeutic value of PUSs and to preliminarily explore the potential mechanisms by which PUSs affect HCC.

## Materials and methods

**FIGURE 1:**
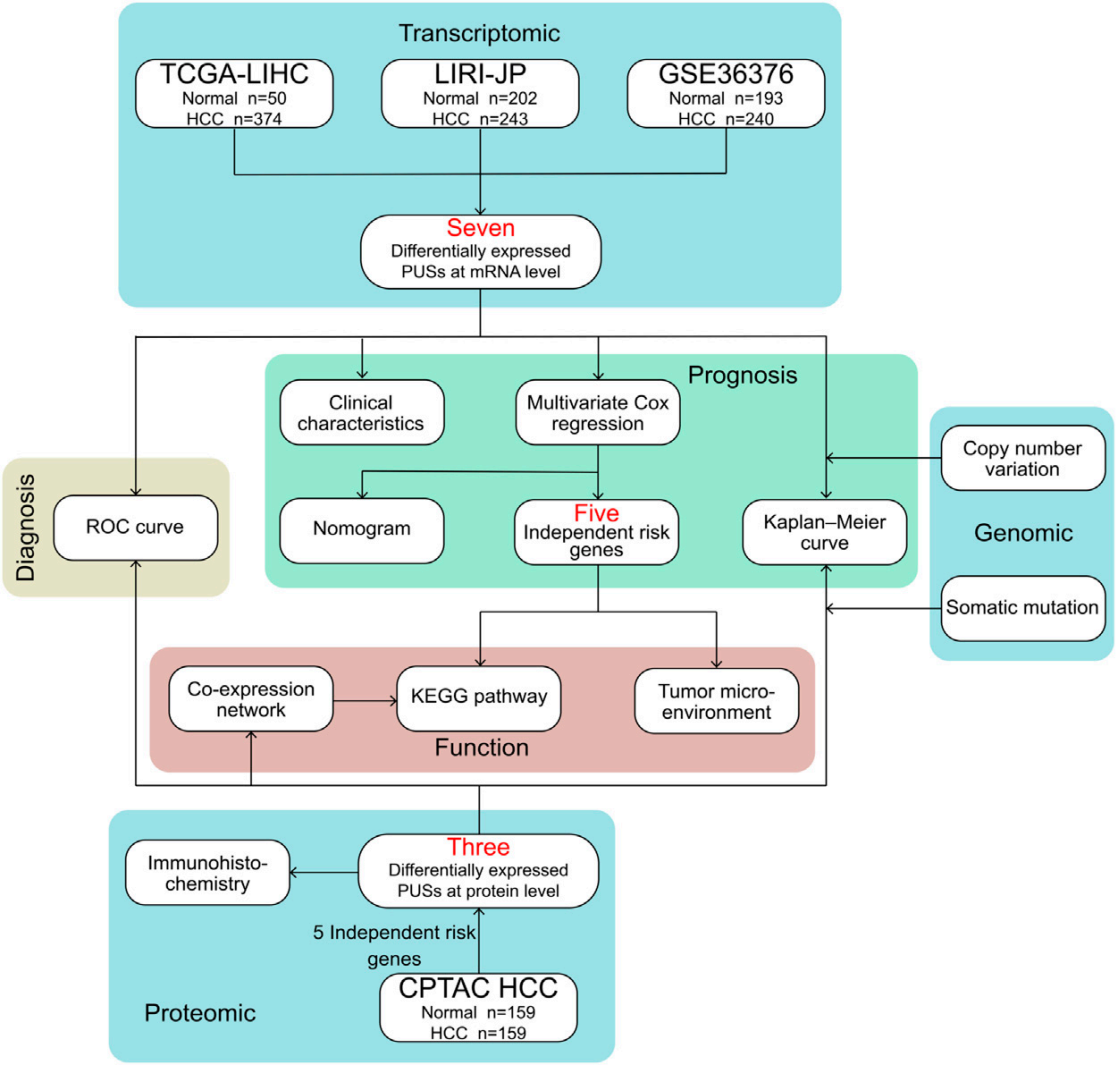
Flowchart of the present research.

The flow chart for this study is shown in Figure 1.

### Dataset sources and processing

Three transcriptomic datasets were analyzed in this study. The TCGA-LIHC (RNA sequencing in FPKM format) cohort, containing 50 adjacent samples and 374 HCC samples, was downloaded from GDC Data Portal (https://portal.gdc.cancer.gov/). The LIRI-JP (RNA sequencing in FPKM format) cohort, containing 202 adjacent samples and 243 HCC samples, was downloaded from ICGC Data Portal (https://dcc.icgc.org/). FPKM values were converted to TPM values for subsequent analyses. Mutation data and clinical data for TCGA-LIHC and LIRI-JP were derived from the same sources as the corresponding expression data. GSE36376 (standardized expression data) cohort, containing 193 adjacent samples and 240 HCC samples, was downloaded from the GEO database (http://www.ncbi.nlm.nih.gov/geo/). The GPL10558 platform file was used as a reference for gene annotation. The average expression of probes was used as the expression value of the gene matched to multiple probes. Expression data for GSE36376 were eligible for direct analysis. The original proteomics and clinical data for an HBV-related HCC cohort (ID: PDC000198) were downloaded from CPTAC Data Portal (https://pdc.cancer.gov/pdc/). The data preprocessing process and the normalized proteomics matrix were obtained from Gao’s study. (Gao et al., 2019). And a total of 6,478 proteins in 159 paired samples were included in subsequent analyses.

### Public online bioinformatics databases

Gene Set Cancer Analysis (GSCA) is a comprehensive database for genomic and immunogenomic cancer analysis (http://bioinfo.life.hust.edu.cn/GSCA/#/) (Liu et al., 2018). Using the GSCA database, we analyzed CNV data from the TCGA-LIHC cohort, including the composition of CNV, the correlation between copy number and gene expression, and the differences in survival of patients with different types of CNV.

The Human Protein Atlas (HPA) is an open-access resource for human proteins (https://www.proteinatlas.org/) (Uhlén et al., 2015). In our study, we assessed the expression levels of PUSs in clinical specimens and obtained several representative immunohistochemical staining results from the HPA database.

### Functional and pathway enrichment analysis

Gene set enrichment analysis (GSEA) was used to identify KEGG pathways related to *PUSs* in HCC tissues (Subramanian et al., 2005). The annotation file (c2.cp.kegg.v7.5.1.symbols.gmt) was obtained from the MSigDB (http://www.gsea-msigdb.org/gsea/downloads.jsp). The analysis was performed using GSEA software (V4.2.1), and the number of permutations was set to 1,000. A false discovery rate (*FDR*) < 0.05 was considered a significant pathway enrichment. Gene set variation analysis (GSVA) was used to estimate the activity of KEGG pathways in each HCC sample (Hänzelmann et al., 2013). The *GSEABase* package was used to read pathway information, and the *GSVA* package was used to convert the gene expression matrix into a gene set score matrix. Moreover, the clusterProfiler package was used to identify and visualize the GO terms (including biological process, cellular component, and molecular function) and KEGG pathways enriched for proteins.

### Correlation of *PUSs* with tumor microenvironment

A previous study conducted an extensive immunogenomic analysis of over 10,000 tumor samples from 33 different cancer types in the TCGA and identified six immune subtypes spanning cancer tissue types (Thorsson et al., 2019). In our study, we analyzed the differences in PUSs expression in samples of different immune subtypes from the TCGA-LIHC cohort. The abundance of different immune cell types and the activity of immune functional pathways in HCC samples were estimated by single sample gene set enrichment analysis (ssGSEA) (Barbie et al., 2009). The analysis was performed using the *GSEABase* package and the *GSVA* package. The ESTIMATE algorithm could score tumor purity, the level of stromal cells, and the level of immune cell infiltration in the tumor tissue based on expression data (Becht et al., 2016). We obtained stromal score, immune score, ESTIMATE score, and tumor purity for HCC samples using the estimate package.

### Collection of the clinical specimens

The clinical specimens involved in this study were obtained from HCC patients who underwent hepatectomy at Qingdao Municipal Hospital. All patients had been pathologically confirmed and diagnosed with HCC. Cancer tissues and matched paracancerous tissue were frozen immediately in liquid nitrogen after resection and then stored at −80°C prior to use. The study was approved by the Ethics Committee of the Qingdao Municipal Hospital and conducted following the Declaration of Helsinki.

### Cell culture

Human hepatocyte LO2 cells and human HCC cells (Hep3B, Huh7) were purchased from the Chinese Academy of Sciences Cell Bank (Shanghai, China). The cells were cultured in Dulbecco’s modified Eagle’s medium (DMEM; HyClone, United States) containing 10% fetal bovine serum (FBS; Excellbio, United States) and 1% penicillin - streptomycin (HyClone, United States). All cells were incubated at 37°C under 5% CO2.

### RNA extraction and quantitative real-time PCR

Total RNA was extracted from clinical tissues or cell lines with TRIzol reagent (Tiangen Biotech, China). cDNA was synthesized from total RNA using a PrimeScript RT reagent kit (TaKaRa). SYBR Green assays (TaKaRa) were used to perform the RT-qPCR. *GAPDH* was used as the loading control, and the 2-ΔΔC t method was used to calculate the relative expression of mRNA. The primer sequences are listed in Supplementary Table S2.

### Statistical analysis

Statistical analyses were performed using R software (V4.1.2). Comparisons between two groups and multiple groups were presented *via* Wilcoxon rank-sum test and the Kruskal–Wallis test, respectively, unless otherwise specified. ROC curves were performed to measure the specificity and sensitivity of the variables for the diagnosis of HCC. Spearman correlation test was adopted to ascertain the correlation between variables. K-M survival curve and the log-rank test were utilized to compare survival differences between groups of patients. The optimal cut-off for groups was determined using the *survminer* package. Univariate and multivariate COX regression was used to perform independent prognostic analysis and to construct a prognostic model. The optimal model was identified based on the Akaike information criterion (AIC). The nomogram was based on the independent prognostic factors filtered by multivariate Cox analysis and generated using the rms package. The predictive performance of the nomogram was evaluated by time-dependent ROC curves and calibration curves. Throughout the analysis, three multivariate COX analyses were performed, which were used for the independent prognostic analysis of single PUS, the construction of the PUS score and the construction of the nomogram, respectively. *p* < 0.05 was considered statistically significant.

## Results

### mRNA expression and diagnostic value of *PUSs* in hepatocellular carcinoma

**FIGURE 2:**
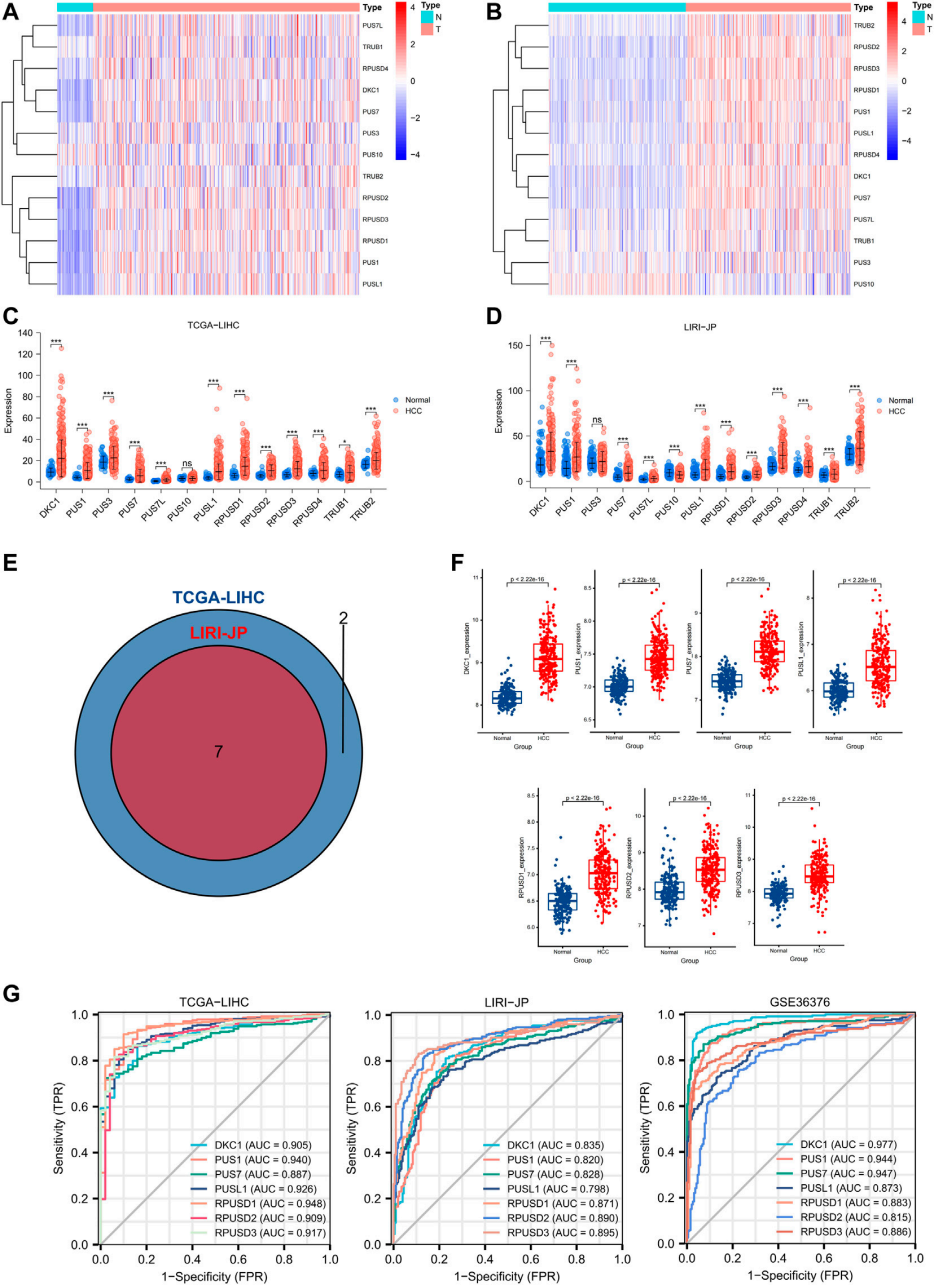
Expression profile and diagnostic value of *PUSs* mRNA in HCC. **(A–B)** Heatmap of *PUSs* expression in TCGA-LIHC **(A)** and LIRI-JP **(B)**; **(C–D)** Expression difference of *PUSs* between non-cancerous tissues and HCC in TCGA-LIHC **(C)** and LIRI-JP **(D)**; **(E)** Venn diagram of differentially expressed *PUSs* in TCGA-LIHC and LIRI-JP; **(F)** Expression of seven differentially expressed *PUSs* between non-cancerous tissues and HCC in GSE36376; **(G)** ROC curves of *PUSs* expression in three HCC cohorts. (ns: no statistical significance, *p < 0.05, **p < 0.01, and ***p < 0.001).

First, we analyzed the mRNA expression levels of 13 *PUSs* in two RNA-seq datasets (Supplementary Table S3). As shown in Figures 2A,B, *PUSs* expression was generally higher in HCC tissues than in non-cancerous tissues. In the TCGA-LIHC cohort, the expression of *DKC1*, *PUS1*, *PUS3*, *PUS7*, *PUS7L*, *PUSL1*, *RPUSD1*, *RPUSD2*, *RPUSD3*, *RPUSD4*, *TRUB1*, and *TRUB2* was upregulated in HCC tissues While there was no difference in the expression of *PUS10* (Figure 2C). In the LIRI-JP cohort, *DKC1*, *PUS1*, *PUS7*, *PUS7L*, *PUSL1*, *RPUSD1*, *RPUSD2*, *RPUSD3*, *RPUSD4*, *TRUB1*, and *TRUB2* were upregulated in HCC tissues, and *PUS10* was downregulated. In addition, the expression of *PUS3* in HCC did not differ from that in non-cancerous tissues (Figure 2D). Next, we calculated the fold change (mean TPM in HCC/mean TPM in normal samples) for each gene and set fold change >1.5 as the threshold. As a result, nine genes were screened in the TCGA-LIHC cohort and seven in the LIRI-JP cohort. Of these, a total of seven genes (*DKC1*, *PUS1*, *PUS7*, *PUSL1*, *RPUSD1*, *RPUSD2*, and *RPUSD3*) met the condition in both cohorts, and we considered them as DEGs (Figure 2E). Then, we validated the expression of DEGs using the GSE36376 cohort, and the trends were consistent with the previous results (Figure 2F). Finally, we evaluated the diagnostic value of DEGs at the mRNA level using ROC curves. As shown in Figure 2G, these genes were of high diagnostic value in all three HCC cohorts (AUC 0.798–0.977).

In summary, we identified 7 *PUSs* that were significantly upregulated in HCC tissues, which may be closely related to HCC and could be used as biomarkers for HCC diagnosis.

### Correlation between *PUSs* expression and clinicopathological parameters

Here, we analyzed the correlation of *PUSs* expression with two important clinicopathological parameters, tumor stage and tumor grade. As shown in Supplementary Figure S1A, in the TCGA-LIHC cohort, all the DEGs had higher expression levels in poorly differentiated tissues than in well-differentiated tissues. In the ICGC cohort, due to incomplete and inaccurate information about tumor grade, we only included part of the samples in the analysis. The results showed that, except for RPUSD2, the median expression of the other six PUSs was higher in poorly differentiated tissues than in well-differentiated tissues (Supplementary Figure S1B). Notably, the results for PUS7, RPUSD2 and RPUSD3 were not statistically significant, which may be due to the small sample size of poorly differentiated cases. Meanwhile, the expression of *DKC1*, *PUS7*, and *RPUSD1* was also higher in patients with advanced HCC (Supplementary Figure S1C). And the correlation between *PUSs* expression and tumor stage was also confirmed in the LIRI-JP cohort (Supplementary Figure S1D). These results suggested that *PUSs* may be involved in the progression of HCC and may be associated with poor prognosis.

### Prognostic value of *PUSs* in hepatocellular carcinoma

**FIGURE 3:**
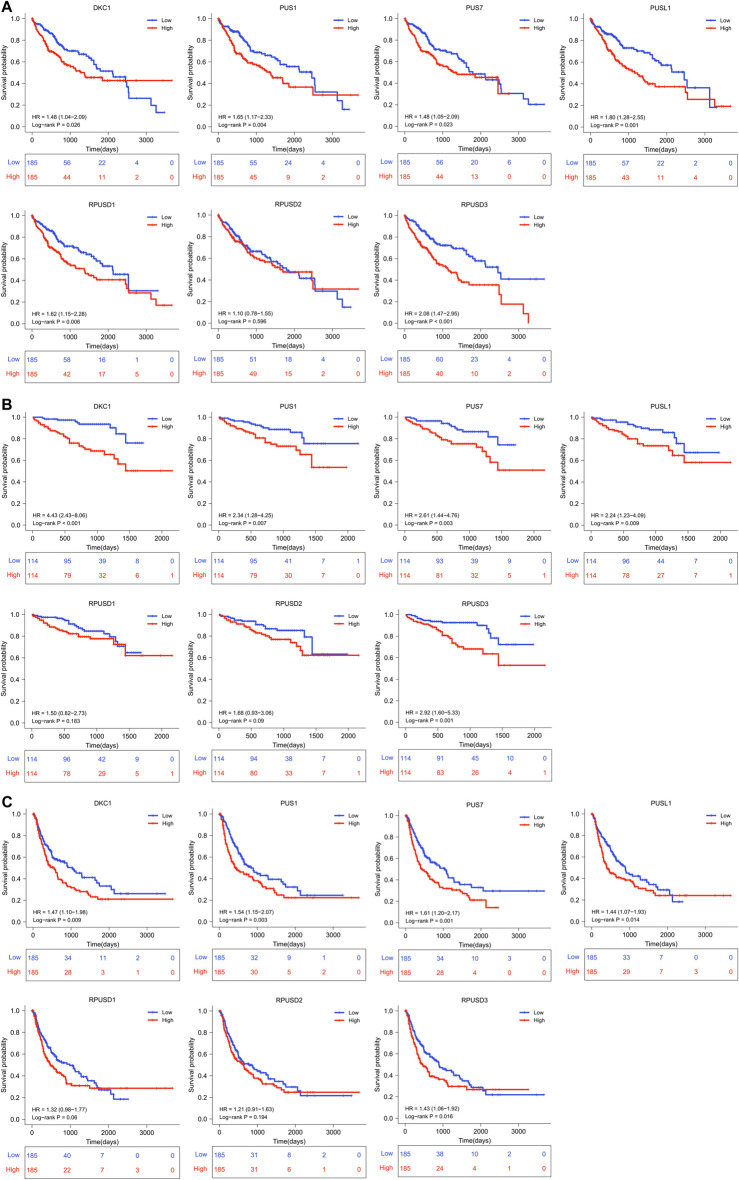
(Continued).

First, we divided all HCC patients into high and low expression groups according to the median level of *PUSs* expression, and analyzed the survival differences between the two groups using the Kaplan-Meier method. As shown in Figures 3A,B, the patients with high expression of *DKC1*, *PUS1*, *PUS7*, *PUSL1*, and *RPUSD3* had better OS. And the same difference was also found in the PFS of patients (Figure 3C).

Then, Cox regression models were used for independent prognostic analysis. We included the four most common clinical features (age, gender, tumor stage, and tumor grade) as confounding factors and excluded the patients who lacked complete clinical information. As a result, a total of 344 patients from the TCGA-LIHC cohort and 228 patients from the LIRI-JP cohort were included in the independent prognostic analysis, respectively (Supplementary Table S4). The univariate analysis showed that *DKC1*, *PUS1*, *PUS7*, *PUSL1*, *RPUSD1*, and *RPUSD3* were associated with poor OS and *DKC1*, *PUS1*, *PUS7*, and *RPUSD3* were associated with poor PFS (Table 1). Further multivariate analysis showed that *DKC1*, *PUS1*, *PUS7*, *PUSL1*, and *RPUSD3* were independent of the above clinical features, and could be used as independent risk factors for OS (Supplementary Figure S2). Meanwhile, *PUS1*, *PUS7*, and *RPUSD3* could be used as independent risk factors for PFS (Supplementary Figure S3). The hazard ratio and 95%CI adjusted by four clinical features were summarized in Table 2. Finally, we validated the expression of these five prognosis-related *PUSs* in clinical tissues and cell lines. As expected, their expression was consistently higher in HCC tissues than in non-cancerous tissues and in HCC cells (Hep3B and Huh7) than in LO2 cells. Besides, the results from the CCLE database (https://portals.broadinstitute.org/ccle/about) showed that these five *PUSs’* expression varied between different hepatocellular carcinoma cell lines (Supplementary Figure S4). These results again demonstrated that *PUSs* might be closely related to HCC.

**TABLE 1:** Univariate Cox analysis of *PUSs* and clinical factors.

Univariate analysis						
Overall Survival (OS)					Progression Free Survival (PFS)	
Characteristics	TCGA-LIHC		LIRI-JP		TCGA-LIHC	
	HR (95% CI)	*P*	HR (95% CI)	*P*	HR (95% CI)	*P*
DKC1	1.021(1.012-1.031)	***<0.001***	1.022(1.014-1.030)	***<0.001***	1.012(1.004-1.021)	***0.003***
PUS1	1.054(1.030-1.080)	***<0.001***	1.023(1.008-1.039)	***0.003***	1.037(1.015-1.060)	***0.001***
PUS7	1.061(1.027-1.097)	***<0.001***	1.073(1.031-1.116)	***<0.001***	1.046(1.017-1.075)	***0.002***
PUSL1	1.026(1.012-1.041)	***<0.001***	1.026(1.007-1.046)	***0.008***	1.005(0.989-1.022)	*0.529*
RPUSD1	1.019(1.005-1.034)	***0.007***	1.037(1.005-1.069)	***0.024***	1.003 (0.989-1.018)	*0.637*
RPUSD2	1.006(0.958-1.055)	*0.823*	1.030(0.939-1.130)	*0.533*	1.032(0.992-1.074)	*0.119*
RPUSD3	1.050(1.024-1.078)	***<0.001***	1.039(1.017-1.062)	***<0.001***	1.033(1.009-1.058)	***0.006***
Age	1.010(0.996-1.025)	*0.174*	1.003(0.973-1.034)	*0.863*	0.994(0.982-1.005)	*0.280*
Gender (M/F)	0.776(0.531-1.132)	*0.188*	0.536(0.287-0.998)	***0.049***	0.933(0.673-1.294)	*0.679*
Grade (G3-4/G1-2)	1.141(0.784-1.661)	*0.490*			1.151(0.841-1.574)	*0.380*
Stage (III-IV/I-II)	2.500(1.721-3.632)	***<0.001***	2.479(1.355-4.533)	*0.003*	2.213(1.596-3.068)	***<0.001***

**TABLE 2:** Results of multivariate Cox analysis for *PUSs*.

Multivariate analysis						
Overall Survival (OS)					Progression Free Survival (PFS)	
Characteristics	TCGA-LIHC		LIRI-JP		TCGA-LIHC	
	HR (95% CI)	*P*	HR (95% CI)	*P*	HR (95% CI)	*P*
DKC1	1.019(1.009-1.028)	***<0.001***	1.018(1.009-1.026)	***<0.001***	1.008(0.999-1.017)	*0.076*
PUS1	1.046(1.021-1.071)	***<0.001***	1.020(1.00 -1.036)	***0.011***	1.030(1.007-1.053)	***0.010***
PUS7	1.056(1.021-1.093)	***0.002***	1.066(1.024-1.110)	***0.002***	1.034(1.003-1.066)	***0.031***
PUSL1	1.018(1.003-1.033)	***0.016***	1.023(1.003-1.043)	***0.024***		
RPUSD1	1.012(0.998-1.027)	*0.104*	1.032(0.998-1.067)	*0.064*		
RPUSD3	1.041(1.015-1.067)	***0.002***	1.032(1.010-1.054)	***0.004***	1.029(1.005-1.054)	***0.016***

### Establishment of a prognostic nomogram based on *PUSs*

To apply the prognostic value of *PUSs* to clinical practice, we further constructed a nomogram using TCGA-LIHC data to predict OS.

**FIGURE 4:**
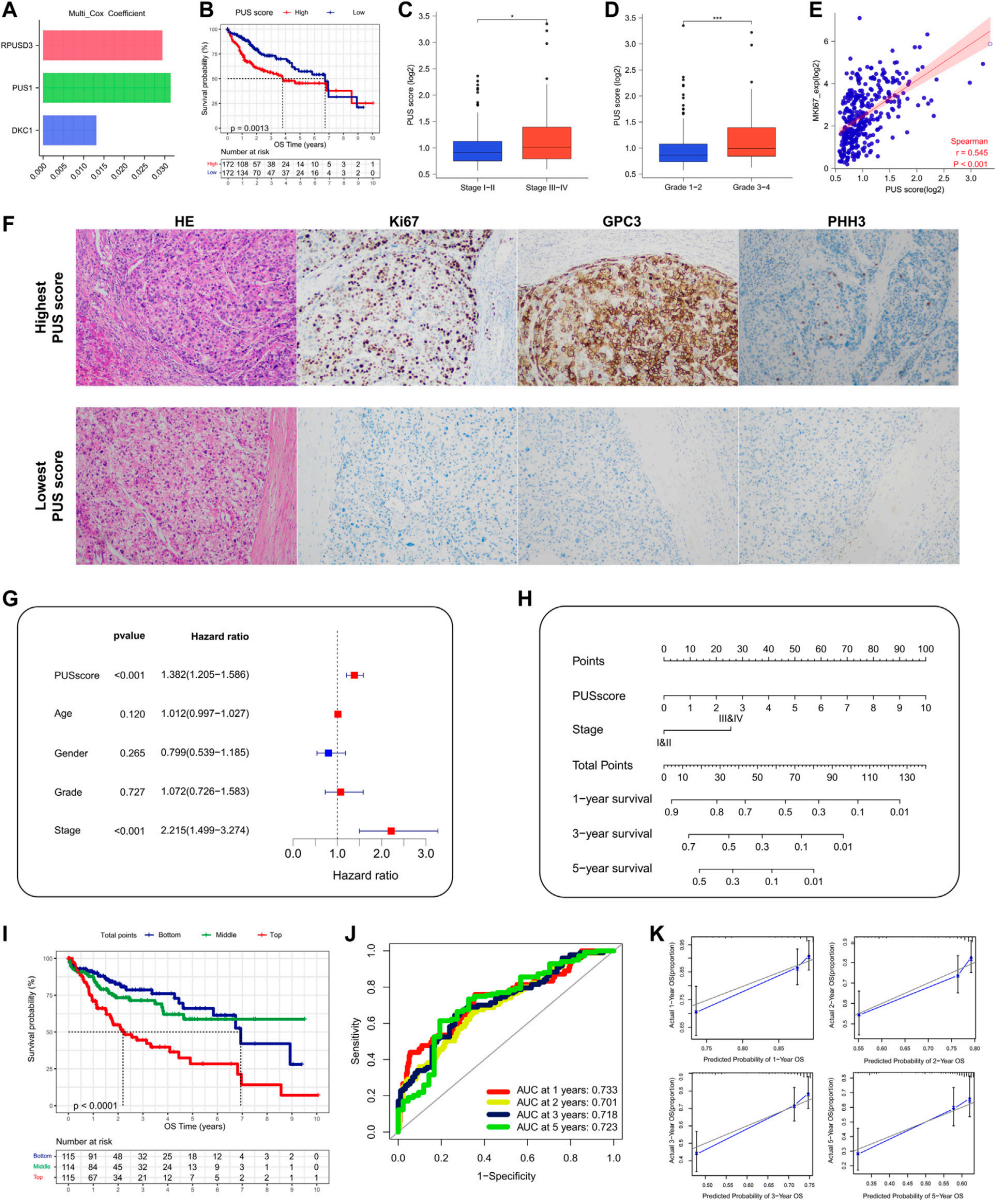
Construction and validation of a *PUS-*based nomogram. **(A)** The coefficients of three optimal *PUSs*; **(B)** K-M analysis for OS of HCC patients stratified by PUSscore; **(C–D)** Association of PUSscore with tumor stage **(C)** and tumor grade **(D)**; **(E)** Correlation of PUSscore with *MKI67* expression; **(F)** Representative HE staining and immunohistochemical results of HCC tissues with different levels of PUSscore; **(G)** Multivariate Cox analysis of PUSscore and clinical factors; **(H)** Development of a nomogram based on PUSscore and tumor stage; **(I)** K-M analysis for OS of HCC patients stratified by nomogram points; **(J)** The time-dependent ROC curves of the nomogram; **(K)** The calibration curves for the nomogram.

First, based on the *PUSs* screened in the univariate analysis, we used multivariate COX analysis again to construct a PUS-related prognostic signature, which was calculated as follows: PUS score = Σ(Expi*coefi). Where Expi and Coefi denote the expression of *PUSs* and the coefficients obtained from multivariate COX analysis, respectively. The coefficients of the 3 *PUSs* in the signature are shown in Figure 4A. As expected, the patients with high PUS scores had poorer OS (Figure 4B). And the PUS score also correlated with the patient’s tumor stage and grade (Figures 4C,D). Besides, we also observed a significant positive correlation between the PUS score and the expression of *MKI67*, the encoding gene of ki67 protein, which is one of the most commonly used prognostic indicators of malignancy in clinical practice (Figure 4E). We then calculated the relative PUS scores for the tissues from our clinical center based on the relative expression of *PUSs*. As shown in Figure 4F, the expression of ki67 was significantly higher in the tissue with the highest PUS score than in that with the lowest PUS score. Further multivariate COX analysis showed that the signature was an independent prognostic factor for OS (Figure 4G). Next, we integrated the PUS score with another prognostic factor, the tumor stage, to establish a nomogram (Figure 4H). With this nomogram, we can calculate the risk score for patients and thus predict their OS. Then, we divided all patients into three groups based on their risk scores, and the K-M curves showed significant differences in OS between the three groups (Figure 4I). The ROC curves showed good accuracy for this nomogram in predicting OS with the AUC was 0.733 for 1-year, 0.701 for 2-year, 0.718 for 3-year and 0.723 for 5-year (Figure 4J), and the calibration curves showed the predictions were almost identical to the actual observations (Figure 4K). These results suggested that the nomogram was a robust prognostic predictor for patients in the TCGA-LIHC cohort. Finally, we validated the above findings in the LIRI-JP cohort (Supplementary Figure S5).

### Copy number variations of *PUSs* and the correlation between *PUSs* and gene mutations in hepatocellular carcinoma

**FIGURE 5:**
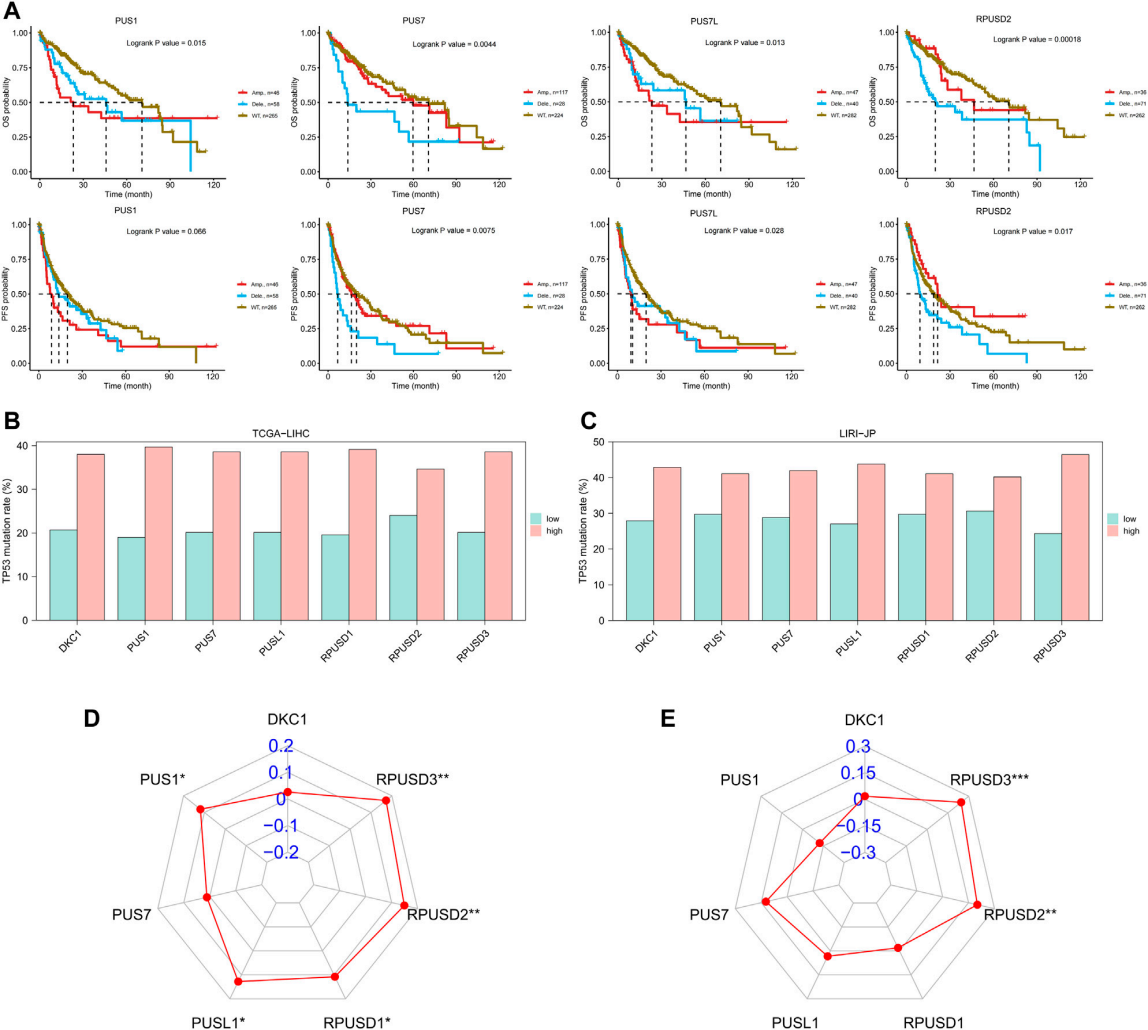
CNVs of PUSs and the correlation between PUSs and mutations. **(A)** Survival differences of HCC patients stratified by *PUSs* CNV; **(B–C)** Differences in TP53 mutation rates in HCC tissues with low versus high *PUS* expression; **(D–E)** Correlation of *PUSs* expression with TMB. (**p < 0.01 and ***p < 0.001).

Here, the CNVs of *PUSs* in the TCGA-LIHC cohort were analyzed by using the GSCA database. First, we obtained the composition of *PUSs* CNVs. As can be seen in Table 3, the proportion of heterozygous CNV was significantly higher than that of homozygous CNV. Among them, *PUS7* had the highest percentage of heterozygous amplification, while *PUSL1* had the highest percentage of heterozygous deletion. Moreover, CNVs of *PUSs* were positively correlated with the mRNA expression of *PUSs*. Then, we analyzed the correlation between CNVs and patients’ prognosis. As a result, CNV of four genes (*PUS1*, *PUS7*, *PUS7L*, and *RPUSD2*) were associated with patients’ prognosis (Figure 5A). Of these, the deletion of *PUS7* and *RPUSD2* copy numbers mean poor OS and PFS. Patients with *PUS1* amplification had the worst OS, and patients with wild type of *PUS1* copy number had the best OS. As for *PUS7L*, its CNV was also associated with a poor prognosis. In detail, compared to *PUS7L* deletion, patients with *PUS7L* amplification had worse OS, but the difference was not significant in PFS. The above results suggested that the CNVs of *PUSs* may also be potential prognostic markers for HCC patients.

**TABLE 3:** Distribution of CNVs of *PUSs* and correlation of CNVs with *PUSs* expression in TCGA-LIHC.

Copy Number Variation of PUSs in TCGA-LIHC								
Gene	Total amplification (%)	Total deletion (%)	Heterozygous amplification (%)	Heterozygous deletion (%)	Homozygous amplificatin (%)	Homozygous deletion (%)	Spearman correlation	FDR
DKC1	23.24	17.57	21.35	17.30	1.89	0.27	0.17	*0.002*
PUS1	12.70	15.68	11.35	15.68	1.35	0.00	0.42	*<0.001*
PUS3	5.95	25.41	5.68	25.14	0.27	0.27	0.34	*<0.001*
PUS7	31.62	7.57	30.54	7.57	1.08	0.00	0.34	*<0.001*
PUS7L	12.97	10.81	12.70	10.81	0.27	0.00	0.28	*<0.001*
PUS10	12.43	9.73	12.16	9.46	0.27	0.27	0.30	*<0.001*
PUSL1	6.22	43.51	5.95	40.54	0.27	2.97	0.36	*<0.001*
RPUSD1	11.35	29.46	10.00	28.92	1.35	0.54	0.39	*<0.001*
RPUSD2	9.73	19.19	9.73	19.19	0.00	0.00	0.43	*<0.001*
RPUSD3	14.05	12.70	12.70	12.70	1.35	0.00	0.36	*<0.001*
RPUSD4	6.22	25.68	5.95	25.41	0.27	0.27	0.39	*<0.001*
TRUB1	9.73	28.65	9.73	28.65	0.00	0.00	0.30	*<0.001*
TRUB2	7.84	30.81	7.30	30.81	0.54	0.00	0.52	*<0.001*

Due to the extremely low incidence of *PUSs* mutation in both HCC cohorts, we did not further analyze its significance. However, we noted a correlation between *PUSs* expression and mutations in *TP53*, the most mutated gene in both HCC cohorts (Supplementary Table S5). Based on the median of 7 DEGs expression, we divided all patients into high and low expression groups. Then we found a higher incidence of *TP53* mutation in the high expression group (Figures 5B,C). We also calculated the TMB of each HCC sample based on the somatic mutation data and found that the expression of *RPUSD2* and *RPUSD3* was positively correlated with TMB (Supplementary Table S6) (Figures 5D,E).

### Potential pathways for the roles of *PUSs* in hepatocellular carcinoma

To further explore the roles of 5 independent prognostic *PUSs* in HCC, we performed functional pathway analyses based on two RNA-seq cohorts.

**FIGURE 6:**
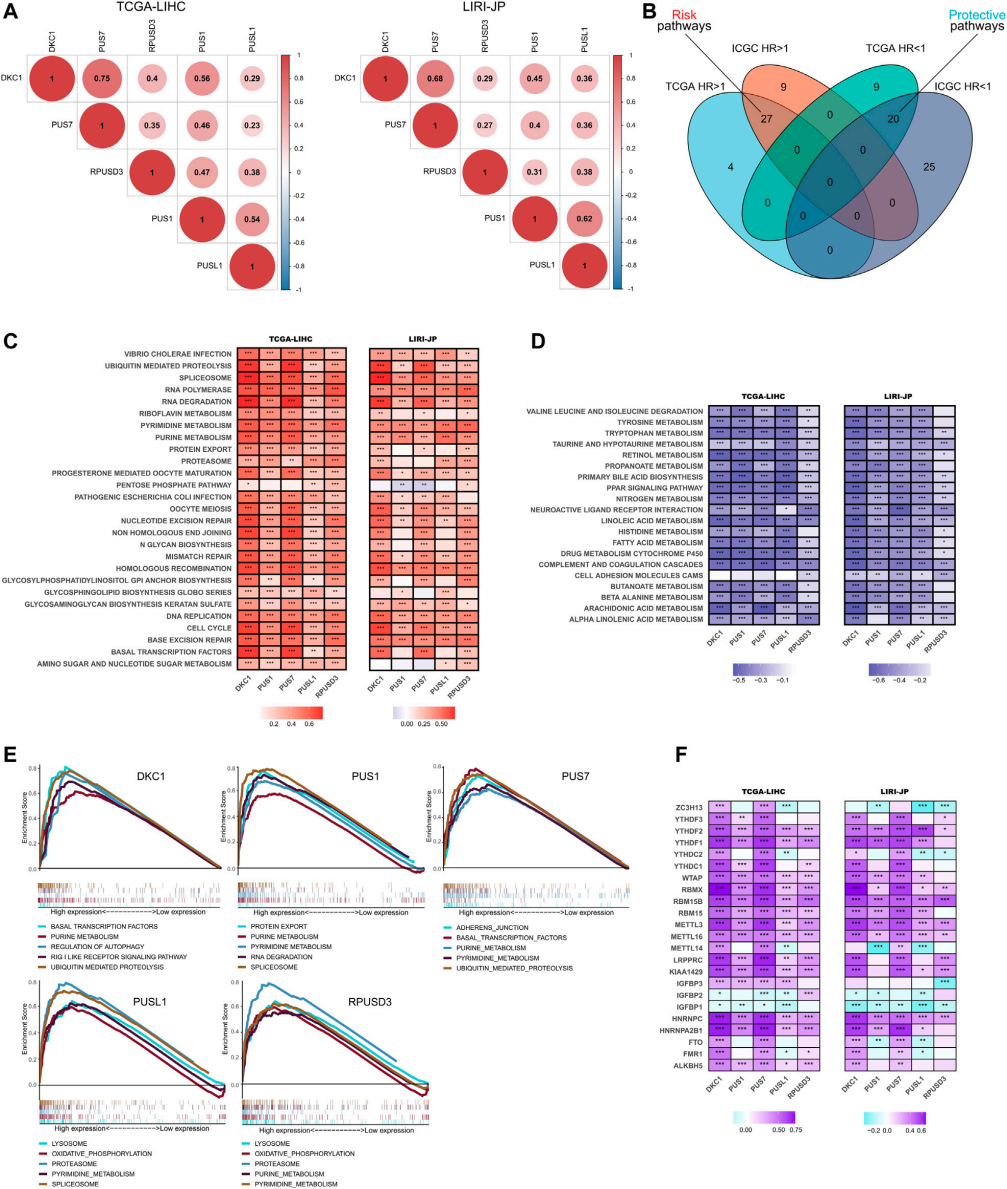
Functional analysis of *PUSs* in HCC. **(A)** Correlation of five prognosis-related *PUSs* expression; **(B)** Venn diagram of prognosis-related pathways (GSVA scores) in TCGA-LIHC and LIRI-JP; **(C–D)** Correlation of *PUSs* expression with GSVA scores of risk pathways **(C)** and protective pathways **(D)**; **(E)** Top five KEGG pathways in high *PUSs* expression group based on GSEA; **(F)** Correlation of *PUSs* expression with m6A regulators expression. (*p < 0.05, **p < 0.01, and ***p < 0.001).

First, we analyzed the correlation between the 5 *PUSs* expression in HCC tissues. As shown in Figure 6A, the expression of these five genes was significantly positively correlated, suggesting that they may act synergistically with each other in HCC.

Next, we performed GSVA to evaluate the enrichment score of 186 KEGG pathways in each HCC sample and used univariate COX models to screen for prognosis-related pathways. As a result, we identified 31 risk pathways (HR > 1, *p* < 0.05) and 29 protective pathways (HR < 1, *p* < 0.05) in the TCGA-LIHC cohort, 36 risk pathways and 45 protective pathways in the LIRI-JP cohort (Supplementary Tables S7, S8). Moreover, 27 risk pathways and 20 protective pathways were filtered as the intersection of two results (Figure 6B). In detail, the risk pathways mainly focused on genetic information processing, substance metabolism, cell cycle, and immune response. And the protective pathways were almost entirely involved in metabolism.

Then, we analyzed the correlation between *PUSs* expression and the enrichment scores of these prognostic KEGG pathways. As expected, the expression of *PUSs* was significantly positively correlated with most risk pathways and negatively correlated with most protective pathways (Figures 6C,D). It was evident that *PUSs* may play broad roles in HCC and lead to poor clinical outcomes. Moreover, we also used GSEA to explore the KEGG pathways that were significantly enriched in samples with high *PUSs* expression (Supplementary Table S9). And the top 5 pathways ranked by NES for each *PUSs* are shown in Figure 6E. Consistent with previous results, many risk pathways were significantly enriched in samples with high *PUSs* expression. In addition, more pathways have been uncovered, including regulation of autophagy, RIG-I-like receptor pathway, adherens junction, lysosome, and oxidative phosphorylation. It can be seen that the relevance of *PUSs* to these pathways cannot be ignored.

Finally, we analyzed the correlation of *PUSs* with m6A, another post-transcriptional modification that is currently being studied with great fervor. As shown in Figure 6F, *PUSs* were significantly correlated with m6A-related genes, which suggested a link between two types of RNA modification, *ψ* and m6A, in HCC.

### Relationship between *PUSs* and tumor microenvironment

**FIGURE 7:**
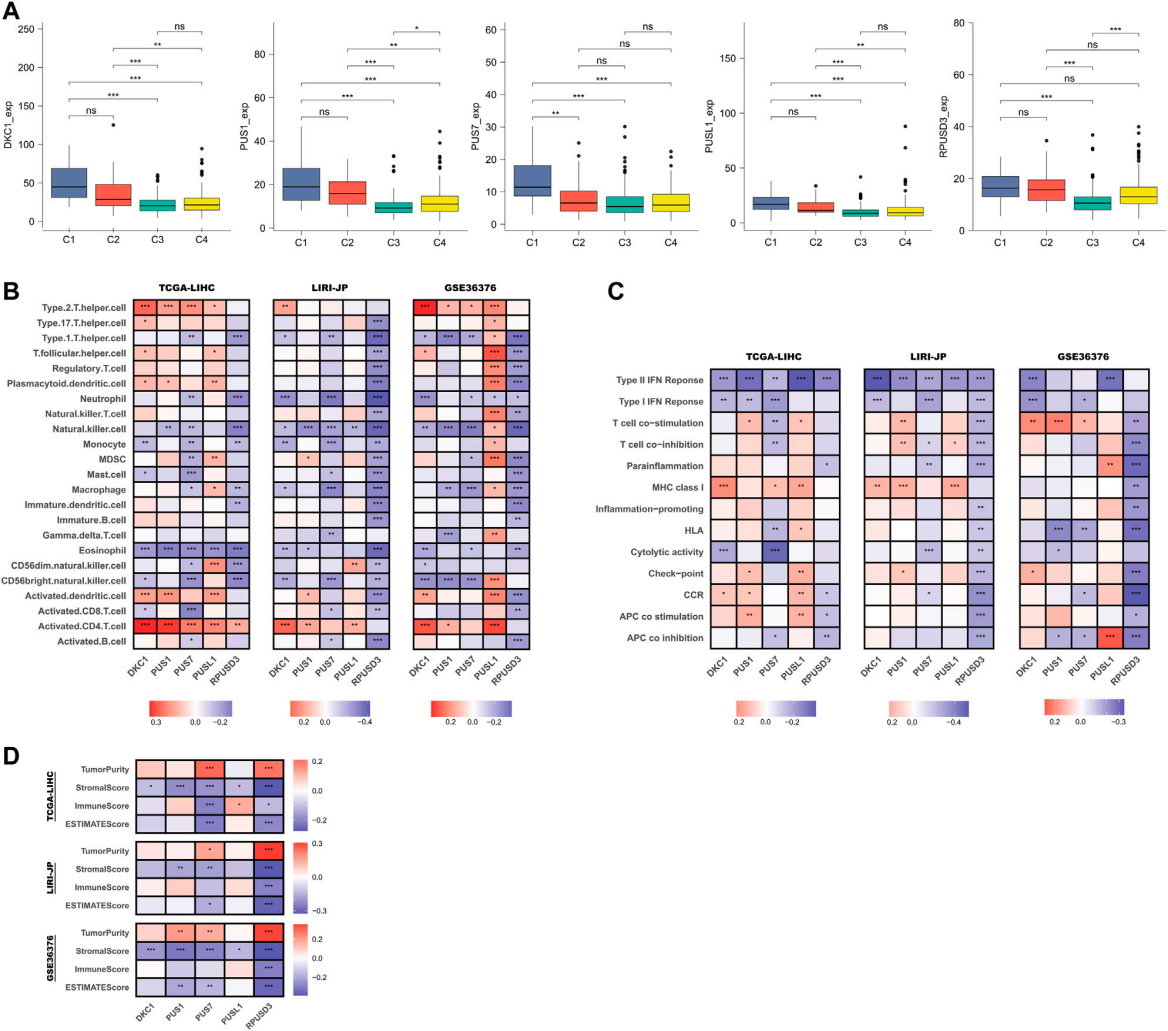
Relationship between *PUSs* and TME. **(A)** Differences in *PUSs* expression in HCC tissues of different immune subtypes; **(B)** Correlation of *PUSs* expression with the estimated abundance of immune cells; **(C)** Correlation of *PUSs* expression with the estimated activity of immune functions; **(D)** Correlation of *PUSs* expression with ESTIMATE score and estimated tumor purity. (ns: no statistical significance, *p < 0.05, **p < 0.01, and ***p < 0.001).

First, we compared the differences in *PUSs* expression in samples of different immune subtypes. In the TCGA-LIHC cohort, there were no samples classified as C5 subtype and, only 1 sample was classified as the C6 subtype (Supplementary Table S10). Therefore, we only compared samples of C1-4 subtypes. As shown in Figure 7A, *PUSs* expression was highest in HCC tissues of the C1 subtype (wound healing), followed by the C2 subtype (IFN-γ dominant). In contrast, *PUSs* expression was lowest in HCC tissues of the C3 subtype (inflammatory). These results suggested that *PUSs* may be associated with the tumor immune microenvironment. Next, we analyzed the correlation between the expression of PUSs and immune cells and immune pathways (Supplementary Tables S11, S12). The results in Figure 7B showed that *PUSs* were associated with multiple immune cell types. For example, the expression of *DKC1*, *PUS1*, and *PUSL1* was positively correlated with the abundance of activated CD4^+^ T cells, *DKC1* expression was positively correlated with Th2 cell, and the expression of *PUS1*, *PUS7*, and *RPUSD3* was negatively correlated with NK cell, etc. Meanwhile, we found that *RPUSD3* was negatively correlated with most of the immune cell types. In addition, *PUSs* were also associated with some immune functions, such as IFN response, T cell co-stimulation (Figure 7C). Notably, *RPUSD3* was also negatively associated with multiple immune functions. Then, we performed the ESTIMATE algorithm to analyze the correlation between *PUSs* and TME (Supplementary Table S13). As shown in Figure 7D, the expression of *PUS1* and *PUS7* was negatively correlated with the stromal score. *RPUSD3* expression was negatively correlated with both stromal and immune scores and positively correlated with tumor purity. Similar to *RPUSD3*, the expression of *PUS7* may also imply higher tumor purity.

### Protein expression of PUSs in hepatocellular carcinoma and their diagnostic and prognostic value

To further explore the role of PUSs in HCC, we also assessed their value at the protein level.

**FIGURE 8:**
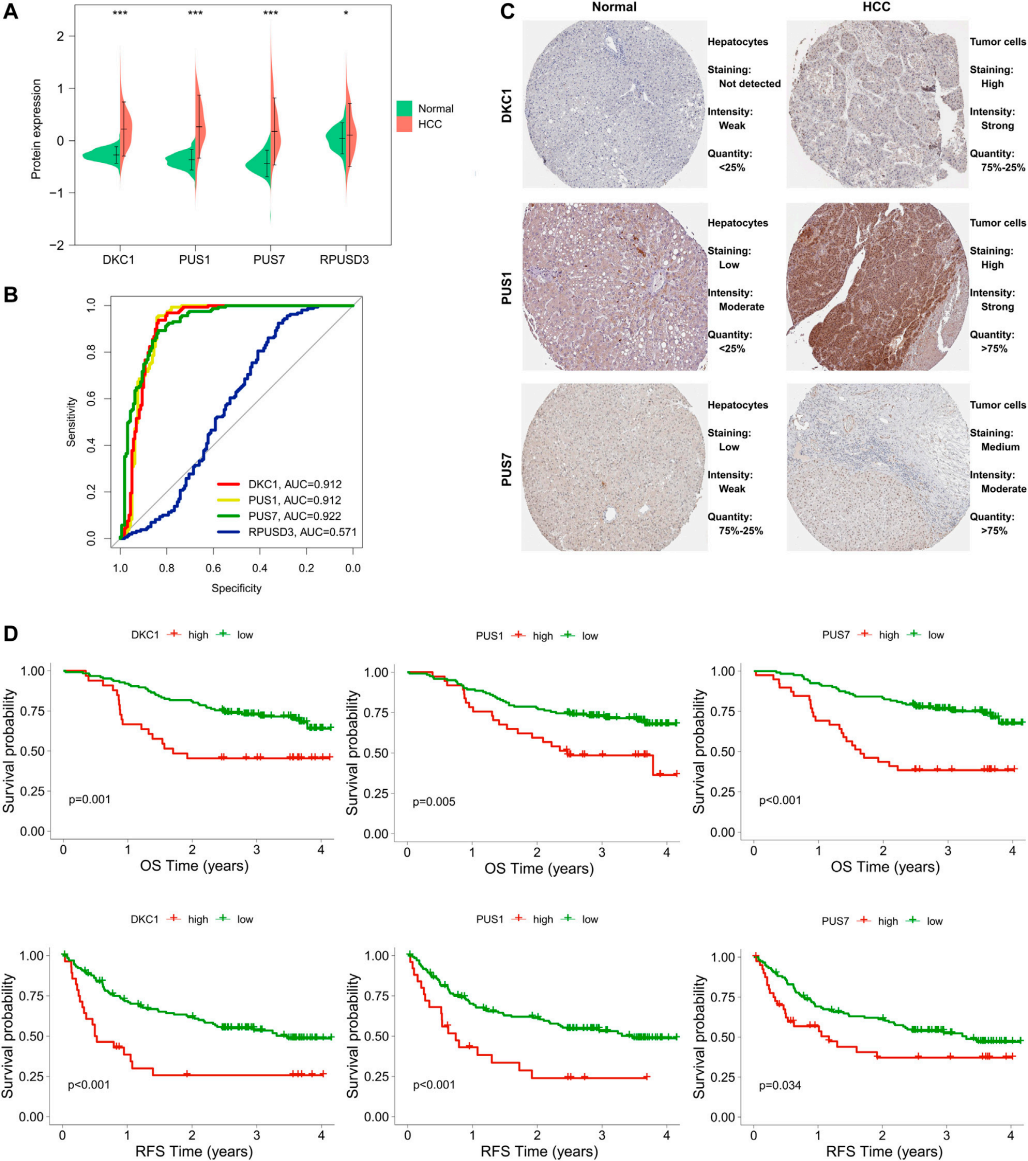
Expression levels of PUS proteins in HCC and their diagnostic and prognostic value. **(A)** Differences in PUS proteins expression between non-cancerous tissues and HCC; **(B)** ROC curves of PUS proteins expression in HCC; **(C)** Representative immunohistochemical results of three differentially expressed PUS proteins; **(D)** K-M analysis for OS and RFS of HCC patients stratified by protein expression of three differentially expressed PUSs. (*p < 0.05, **p < 0.01, and ***p < 0.001).

First, we compared the differences in protein expression levels of these five PUSs in HCC tissues and normal tissues. As a result, four proteins (DKC1, PUS1, PUS7, and RPUSD3) were differentially expressed in the two groups of samples (Figure 8A). Notably, in the raw proteomics data, PUSL1 did not pass the quality control criteria for the data and was therefore excluded from the analysis. To ensure the rigour of the study, we verified the results of PUS protein expression using the UALCAN database (http://ualcan.path.uab.edu/). As expected, DKC1, PUS1, PUS7 and RPUSD3 were significantly upregulated in HCC tissues. In contrast, PUSL1 expression was not significantly different in HCC and normal tissues (Supplementary Figure S6). Next, we evaluated the diagnostic value of these 4 PUSs using ROC curves and found that DCK1, PUS1, and PUS7 (AUC of 0.912, 0.912 and 0.922, respectively) could serve as diagnostic markers for HCC (Figure 8B). The above results suggested that DCK1, PUS1, and PUS7 were closely associated with HCC. Then, we obtained the representative immunohistochemical results from the HPA database, which also confirmed the higher expression of these 3 PUSs in HCC specimens than in normal liver specimens (Figure 8C). Finally, we analyzed the correlation between 3 PUSs and patients’ prognosis using K-M curves. We divided the patients from the CPTAC cohort into two groups according to the best cut-off value of PUSs expression and found that patients in high expression groups had worse OS and RFS (Figure 8D).

Taken together, DKC1, PUS1, and PUS7 were closely related to HCC and may serve as potential biomarkers for the diagnosis and prognosis of HCC.

### Functional enrichment analysis of the PUSs co-expression network

**FIGURE 9:**
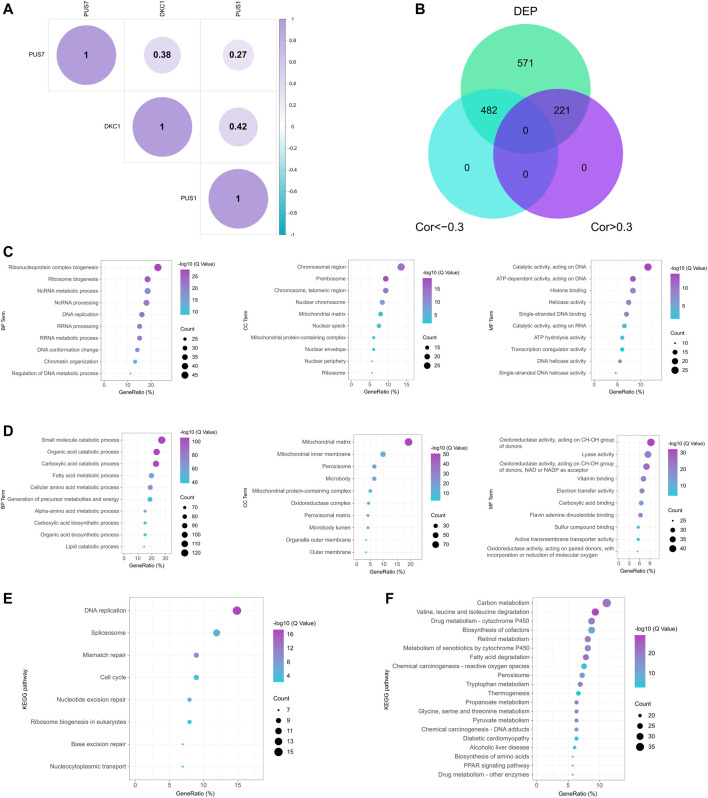
Potential functions of PUS proteins in HCC. **(A)** Correlation of three prognosis-related PUS proteins expression; **(B)** Venn diagram of DEPs co-expressed with PUS proteins; **(C–D)** GO terms for DEPs positively **(C)** and negatively **(D)** correlated with PUS proteins; **(E–F)** KEGG pathways for DEPs positively **(E)** and negatively **(F)** correlated with PUS proteins.

Here, we explored the roles of PUSs in HCC. First, correlation analysis showed that DKC1, PUS1, and PUS7 were co-expressed (Figure 9A). Consistent with the results at the mRNA level, they may play synergistic roles in HCC. Next, we screened for proteins associated with these 3 PUSs. In the CPTAC cohort, we found 1,274 DEPs using fold change >1.5 as the threshold. After that, we used the correlation coefficient to measure their correlation with the PUSs, with |r| > 0.3 being considered significant. As a result, 482 proteins negatively associated with PUSs expression and 221 positively associated with PUSs expression were identified, respectively (Figure 9B). Finally, we analyzed the GO terms and KEGG pathways enriched by these proteins (Supplementary Table S14). The count of enriched genes was used as the ranking criterion, and the top 10 GO terms for both groups of proteins were shown in Figures 9C,D, and the top 20 KEGG pathways were shown in Figures 9E,F. The results showed that the proteins positively associated with PUSs mainly function in genetic information processing (such as DNA replication and damage repair, transcription, and translation) and the cell cycle. Meanwhile, the proteins negatively associated with PUSs mainly function in metabolism. These results were almost identical to those at the mRNA level, which suggested that PUSs may affect HCC precisely by regulating these functions or pathways and gave us an initial insight into the role of PUSs in HCC.

## Discussion

Pseudouridine (*ψ*) was the first post-transcriptional modification discovered and is one of the most abundant RNA modification types (Charette and Gray, 2000). Alterations in its deposition are involved in human diseases, including cancers (Nombela et al., 2021). For example, high levels of *ψ* have been detected in body fluids of patients with colon, prostate, ovarian and oral squamous cell carcinomas, suggesting that *ψ* could be a potential biomarker for cancer diagnosis (Jiang et al., 2015; Krstulja et al., 2017; Sridharan et al., 2019; Stockert et al., 2019; Zeleznik et al., 2020). This has led to an increasing interest in the role of its modifying enzymes in cancer. Unsurprisingly, PUS is indeed associated with many human cancer types (Nombela et al., 2021). However, few studies have explored the role of PUS in HCC. To fill this scientific gap, our study integrated transcriptomic, proteomic and genomic data from HCC patients and confirmed the importance of PUS in HCC.

Previous studies have found that both protein and mRNA expression of DKC1 was higher in HCC than in non-cancerous liver tissues and was associated with advanced clinical stage and poor prognosis (Liu et al., 2012; Ko et al., 2018; Zhang et al., 2021b; Li et al., 2022a). As for RNA-independent PUSs, their roles in HCC have rarely been explored. A previous study constructed a lactate metabolism-related gene signature to predict the prognosis of HCC patients, and *PUS1* was a key risk factor in this gene signature (Li et al., 2022b). To our knowledge, this is the only study that explores the value of RNA-independent PUSs in HCC. In our study, we found that seven PUS genes (*DKC1*, *PUS1*, *PUS7*, *PUSL1*, *RPUSD1*, *RPUSD2,* and *RPUSD3*) were significantly upregulated in HCC and they were expressed at higher levels in advanced stage or poorly differentiated HCC tissues. Importantly, all these 7 *PUSs* can be used as diagnostic markers for HCC, and 5 of them (*DKC1*, *PUS1*, *PUS7*, *PUSL1*, and *RPUSD3*) were risk factors for patients’ survival independently of age, gender, stage and grade. Moreover, the protein levels of DKC1, PUS1, and PUS7 were also significantly upregulated in HCC and associated with poor prognosis. Our findings filled a gap where most PUSs have been left unstudied in HCC and confirmed the involvement of PUSs in HCC. The nomogram we further constructed also applied the prognostic value of *PUSs* to the clinic, providing clinicians with a guide that can accurately predict patient prognosis.

As for the molecular mechanism of PUS involvement in cancer, it has been reported that overexpression of *DKC1* increased the expression of TERC and rRNA pseudouridylation, promoting the proliferation of colorectal cancer (Turano et al., 2008; Nersisyan et al., 2019). The upregulation of PUS7 promoted CRC cell metastasis and was independent of the catalytic activity of PUS7. This mean PUS7-mediated *ψ* may not govern the metastatic capacity of CRC cells. Meanwhile, the HSP90/PUS7/LASP1 axis was a potential mechanism by which PUS7 promoted CRC metastasis (Song et al., 2021). Another study showed that PUS7 could promote CRC cell proliferation and invasion by activating PI3K/AKT/mTOR signaling pathway (Du et al., 2021). In glioma, *DKC1* up-regulation was common and necessary for extensive tumor growth. *DKC1* could upregulate the expression of N-cadherin, MMP-2, HIF1A, CDK2, and cyclin E, and the glioma cells with *DKC1* knockdown exhibited low motility. Meanwhile, *DKC1* knockdown also altered the expression of cell cycle-relative molecules to arrest at the G1 phase (Miao et al., 2022). Moreover, the molecular mechanisms of PUS1 involvement in melanoma and breast cancer, and PUS10 involvement in prostate cancer have also been initially explored (Zhao et al., 2004; Jana et al., 2017).

So how is PUS involved in HCC? What functions are available? What pathways? Unfortunately, there has not been sufficient research to address these issues. As for some typical pathways with strong relevance to PUS *i*n our study, abnormalities in cell cycle progression are one of the fundamental mechanisms of tumorigenesis. Cell cycle regulatory pathways are combined with other features of cancer, including metabolic remodelling and immune escape. This makes regulators of the cell cycle machinery a reasonable target for anti-cancer therapy (Liu et al., 2022). Meanwhile, the cell cycle is driven by the activation of cell cycle protein-dependent kinases (CDKs), whose activity is controlled by ubiquitin-mediated proteolysis of key regulators such as cell cycle proteins and CDK inhibitors (Nakayama and Nakayama, 2006). From this, we inferred that ubiquitin-mediated proteolysis may be closely related to the cell cycle, and they might synergistically or independently affect the prognosis of HCC patients. Next, we turn our attention to transcription-related pathways. The spliceosome is thought to be general cellular ‘housekeeping’ machinery. Spliceosomal mutations and aberrant splicing are also closely associated with human cancer. Also, some spliceosome gene mutations can lead to immune dysregulation. As a result, spliceosome-targeted therapies (STTs) have emerged as highly effective anti-cancer strategies (Yang et al., 2021). Transcription mediated by RNA polymerases is an important factor in determining the growth of cancer cells. However, inactivation of some tumor suppressors (e.g., p53) in cancer can dysregulate RNA polymerases, and oncoproteins (e.g., Myc) can further stimulate these systems. Such events may have a significant impact on the growth of cancer cells (White, 2005). And the basal transcription factors play a vital role in the initiation of transcription of the encoded genes. They are a group of protein molecules necessary for RNA polymerase to bind the promoter (Reese, 2003). As for DNA replication and damage repair, their involvement in cancer and as features of cancer is an accepted fact and therefore does not require more elaboration (Modrich, 1994; Gaillard et al., 2015; Jeggo et al., 2016; Carbone et al., 2020). Cancer is also a metabolic disease due to a metabolic disorder (Boroughs and DeBerardinis, 2015). Then HCC is destined to be inextricably linked to metabolic disorders, as the liver is the central metabolic organ of the body (Ng et al., 2017). The prognosis-related pathways derived in our study again supported the idea that metabolism may be another determinant of the impact of PUSs on HCC. Interestingly, the effect of PUSs on nucleotide metabolism appeared to be distinct from its effect on the metabolism of other substances, although they both ultimately lead to a poorer prognosis. Overall, the pathways regulated by PUSs in HCC are likely to be very broad and interconnected. And it is also unknown whether these functions are dependent on the catalytic activity of PUS. In future, research should focus on the effect of PUS on the above pathways in HCC.

TME also plays crucial roles in the pathogenesis, malignant features, and treatment of HCC (Hernandez-Gea et al., 2013). Therefore, the link between PUSs and TME should also be considered. The correlation between cell cycle and immune response, the enrichment of immune-related pathways in GSEA, the differential expression of PUSs in different immune subtypes of tissues, the correlation between PUSs expression and immune cell infiltration as well as immune function, and the association of PUSs expression with the ESTIMAT score, they all suggested that PUSs may be potential regulators of TME. Among them, the expression of *RPUSD3* seemed to imply a lack of immune cells and a low level of many immune functions in the TME. This was supported by the positive correlation between its expression and tumor purity. So what does the association of *PUSs* with TME lead to? Some examples, it was found that NK cell was the main antitumor cell type in the liver (Sonnenberg and Hepworth, 2019). The IFN response plays crucial roles in promoting host antitumor immunity and is considered a critical component of the cancer elimination phase of cancer immunosurveillance (Dunn et al., 2006). Therefore, we hypothesized that the role of PUSs in HCC may be partly through influencing antitumor immunity. Nevertheless, the TME is very complex. Some ingredients can even have opposite effects under different conditions. The mechanisms by which PUSs shape the TME are yet to be further explored.

There is now consensus that mutation of TP53 converts it from a tumor suppressor gene to an oncogene. The mutated TP53 loses its original regulatory functions, which are mainly focused on cell cycle, apoptosis, DNA repair, etc. (Vaddavalli and Schumacher, 2022). This also coincides with the findings of our study. As for the CNV of *PUSs*, its impact on the prognosis of HCC patients has also been revealed. Hence, more emphasis should be placed on the CNV of *PUSs* in future.

Certain limitations of our study are to be acknowledged. First, due to data limitations, some critical risk factors, such as HBV infection, were not included in the analysis, which may have led to biased conclusions. Second, the proteomic analysis was based on only one Chinese HBV-related HCC cohort. Therefore, there is a need to further dissect the value of PUS proteins in a larger sample. Third, the molecular mechanisms underlying the role of PUSs in HCC need to be further investigated through comprehensive *in vivo* and *in vitro* experiments. We will continue to focus on these issues in future research.

## Conclusion

In conclusion, multiple PUS genes expression was upregulated in HCC. Among them, *DKC1*, *PUS1*, *PUS7*, *PUSL1*, and *RPUSD3* were independent risk factors for OS. At the protein level, the expression of DKC1, PUS1, and PUS7 was upregulated in HCC and correlated with poor prognosis. Moreover, both mRNA expression and protein expression of PUSs were highly diagnostic of HCC. Further functional analysis revealed that PUSs might be involved in the regulation of multiple pathways in HCC. Therefore, PUSs may play essential roles in HCC and can be used as potential biomarkers for the diagnosis and prognosis of patients.

## Data Availability

The datasets presented in this study can be found in online repositories. The names of the repository/repositories and accession number(s) can be found in the article/Supplementary Material.

## Supplementary material

SUPPLEMENTARY FIGURE S1 Correlation between PUSs expression and clinicopathological parameters.

SUPPLEMENTARY FIGURE S2 Multivariate Cox analysis of PUSs mRNA and clinical factors for OS.

SUPPLEMENTARY FIGURE S3 Multivariate Cox analysis of PUSs mRNA and clinical factors for PFS.

SUPPLEMENTARY FIGURE S4 Relative expression of five prognosis-related PUSs in clinical tissues and cell lines.

SUPPLEMENTARY FIGURE S5 Validation of PUS score and nomogram in the LIRI-JP cohort.

SUPPLEMENTARY FIGURE S6 Results of differential analysis of PUS protein expression in the UALCAN database.

SUPPLEMENTARY TABLE S1 Thirteen pseudouridine synthases in human cells;

SUPPLEMENTARY TABLE S2 Primer sequences used for RT-qPCR.

SUPPLEMENTARY TABLE S3 Results of differential expression analysis of PUS mRNA.

SUPPLEMENTARY TABLE S4 Patients involved in multivariate Cox analysis.

SUPPLEMENTARY TABLE S5 TP53 mutation status of HCC samples.

SUPPLEMENTARY TABLE S6 TMB data of HCC patients.

SUPPLEMENTARY TABLE S7 GSVA scores of the KEGG pathways in HCC samples.

SUPPLEMENTARY TABLE S8 Univariate Cox analysis of KEGG pathways for OS.

SUPPLEMENTARY TABLE S9 GSEA results for high PUSs expression samples in TCGA-LIHC.

SUPPLEMENTARY TABLE S10 Immune subtype of HCC samples in TCGA-LIHC.

SUPPLEMENTARY TABLE S11 The annotated gene sets used in ssGSEA.

SUPPLEMENTARY TABLE S12 Results of ssGSEA.

SUPPLEMENTARY TABLE S13 Results of ESTIMATE.

SUPPLEMENTARY TABLE S14 Results of the functional enrichment analysis of proteins.
