# Supplementary figures and images for: Integrative multiomics evaluation reveals the importance of pseudouridine synthases in hepatocellular carcinoma
Source: Front Genet. 2022 Nov 10;13:944681. doi: 10.3389/fgene.2022.944681 (PMC9686406; doi:10.3389/fgene.2022.944681)

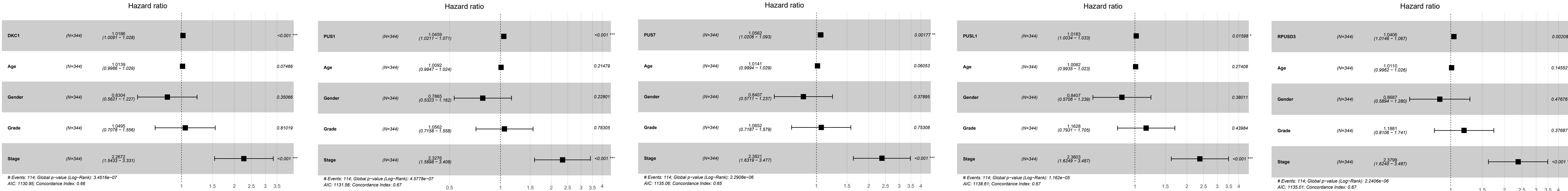

Supplement: Supplementary file 1 [file DataSheet2.PDF]

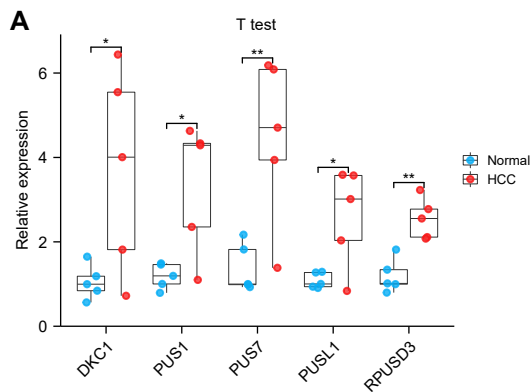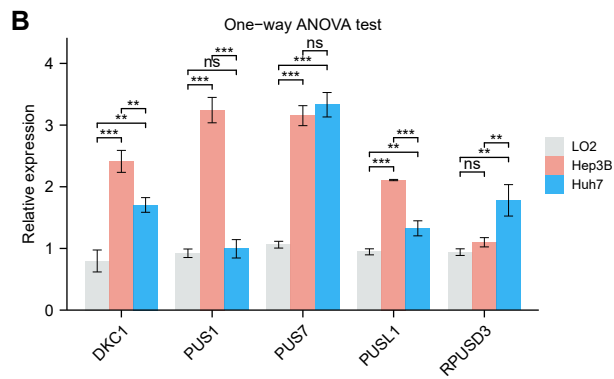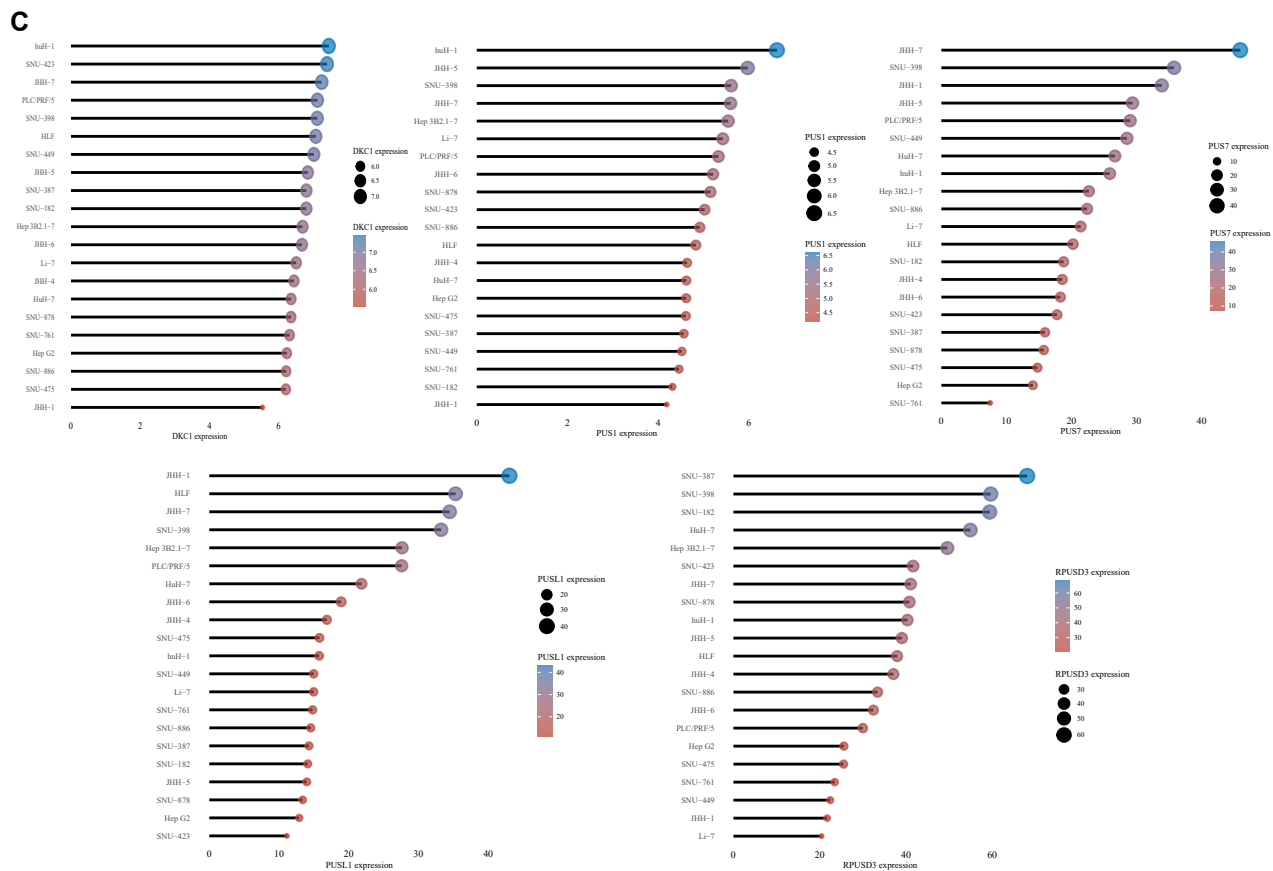

Supplement: Supplementary file 4 [file DataSheet4.PDF]

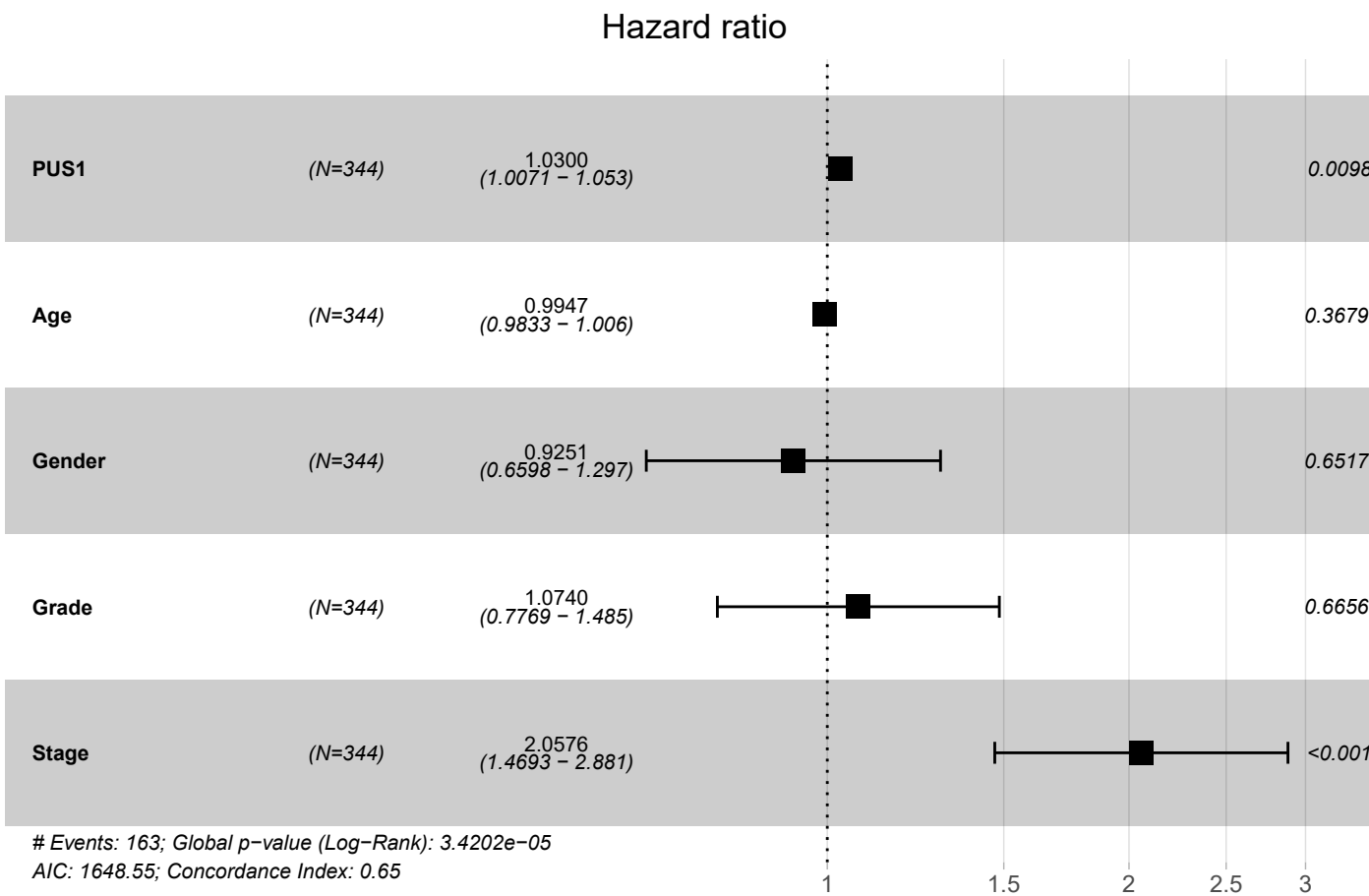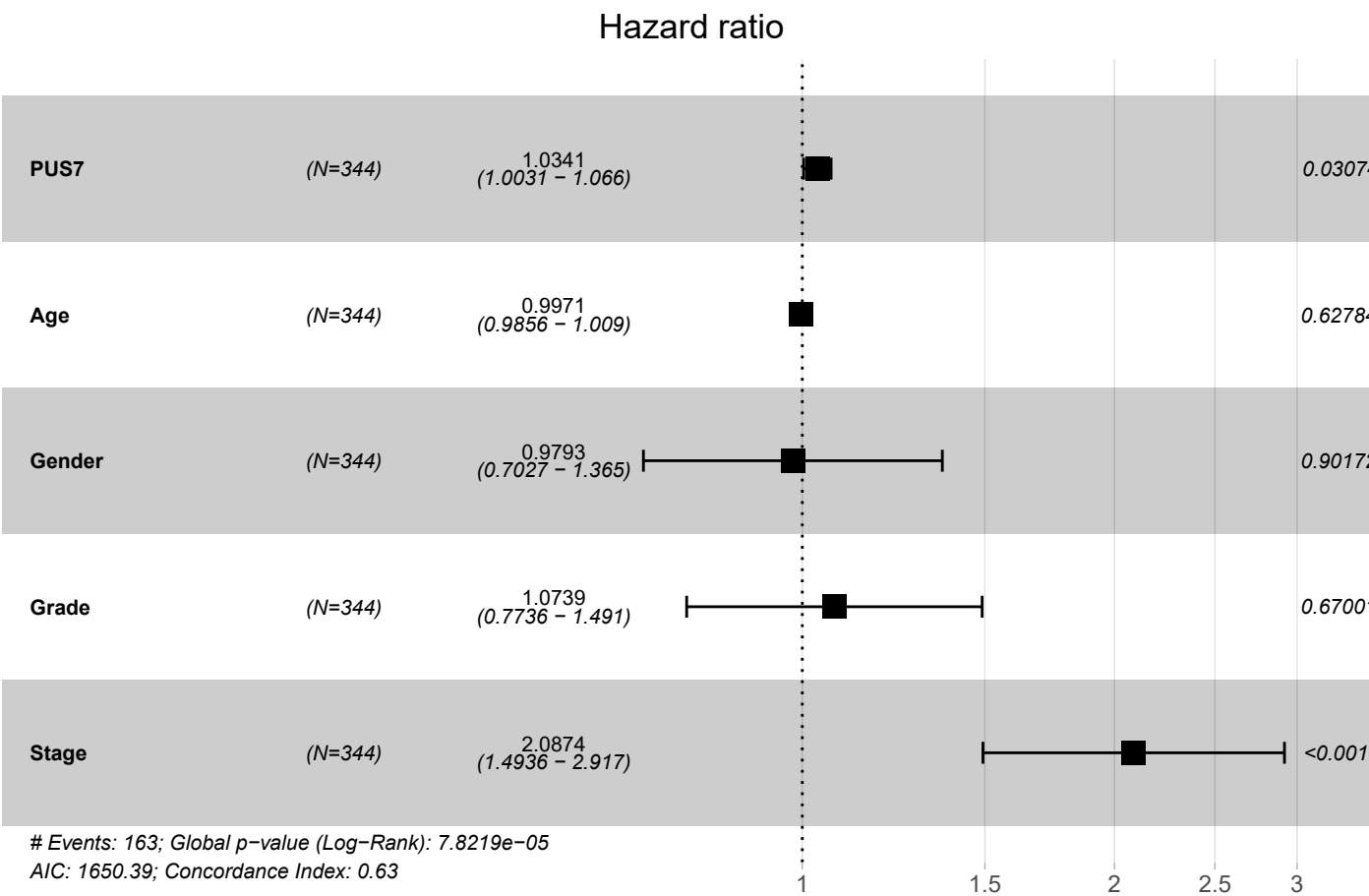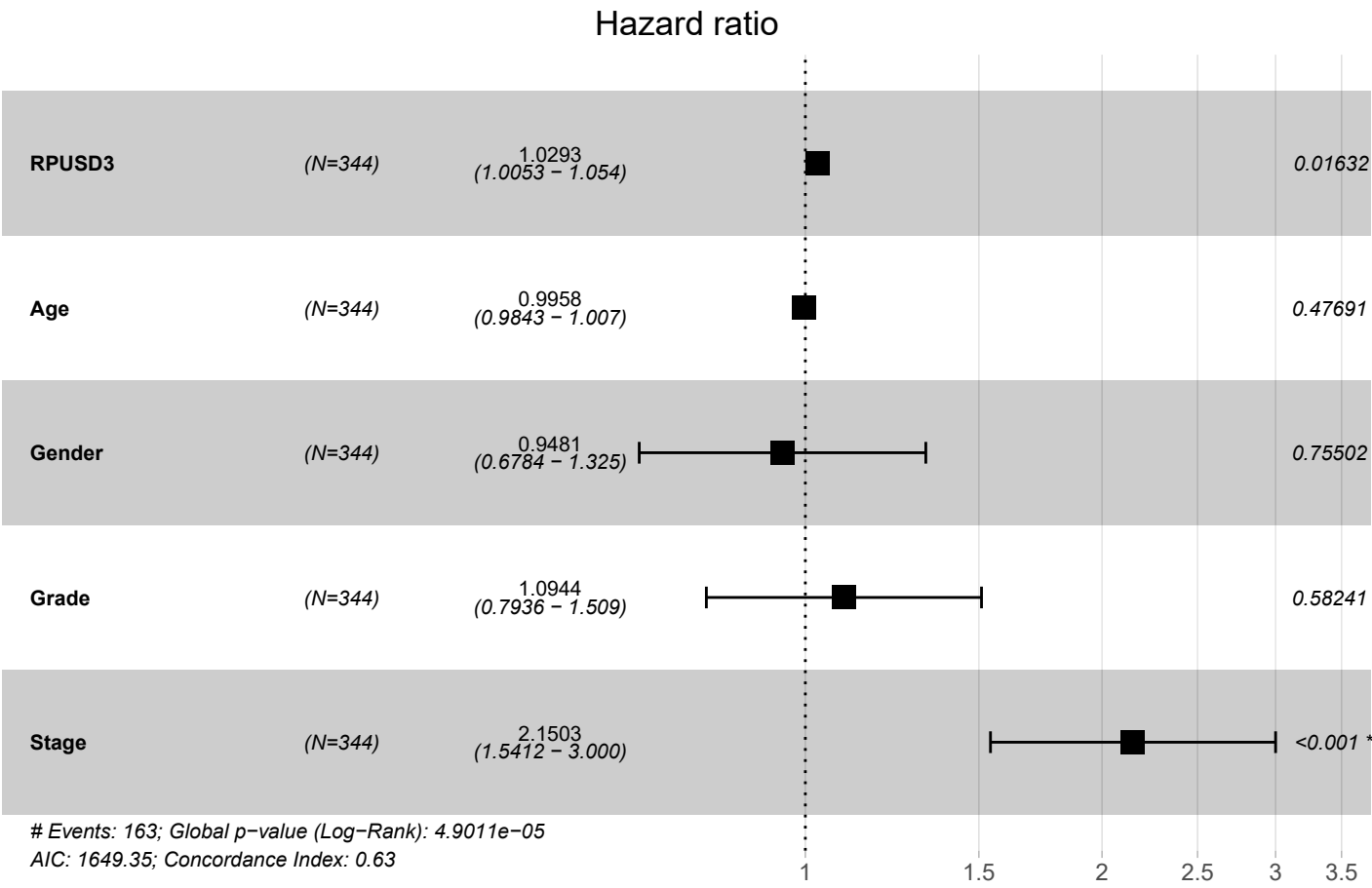

Supplement: Supplementary file 8 [file DataSheet3.PDF]

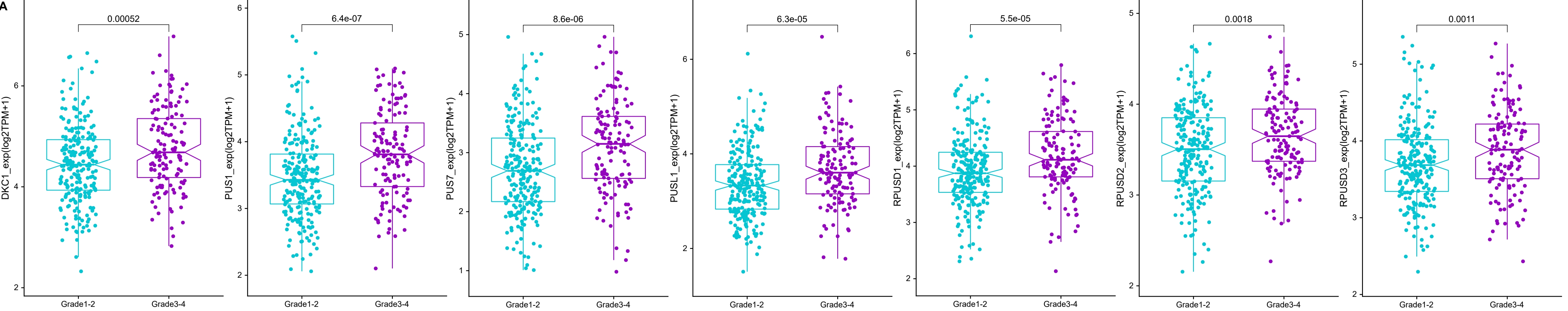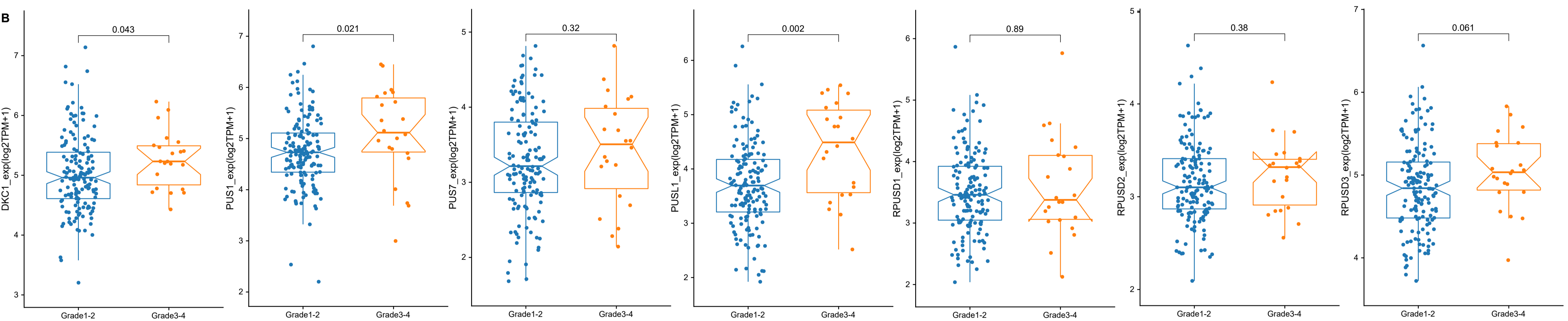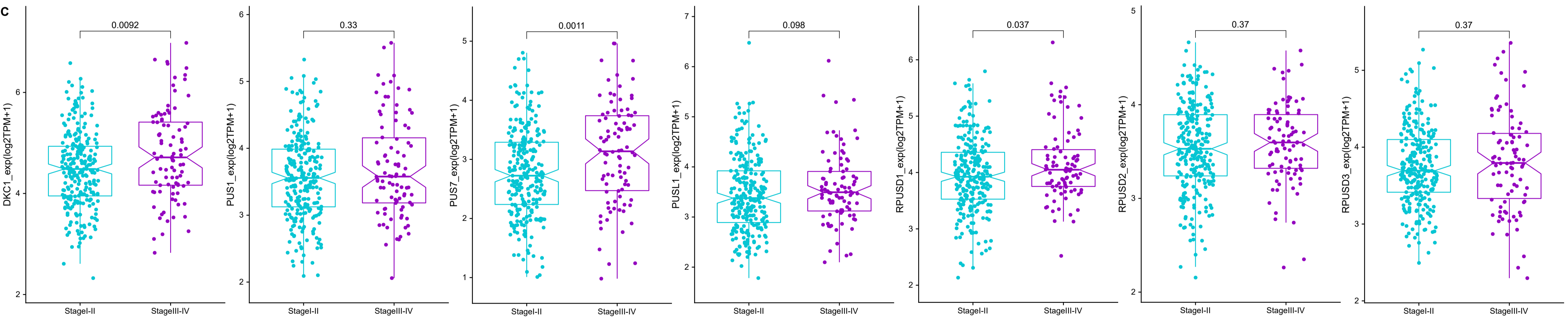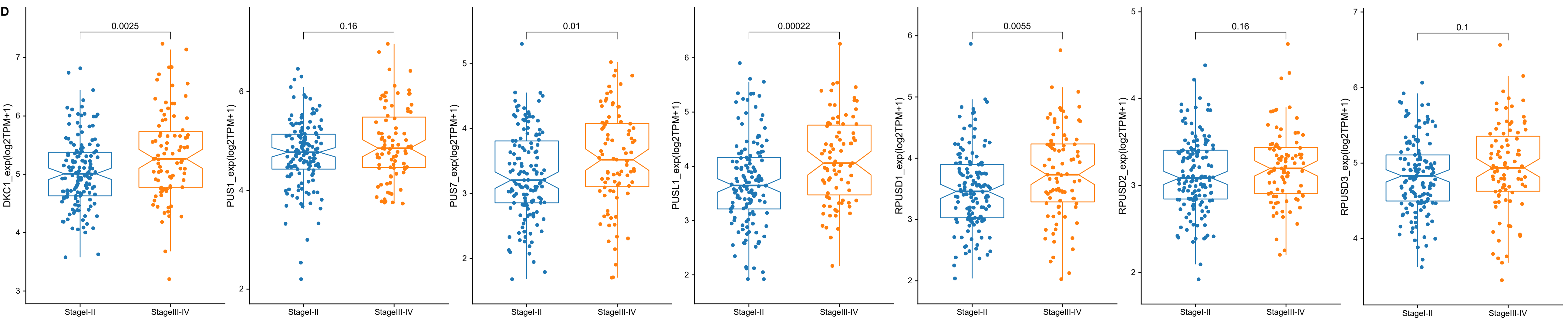

Supplement: Supplementary file 10 [file DataSheet1.PDF]

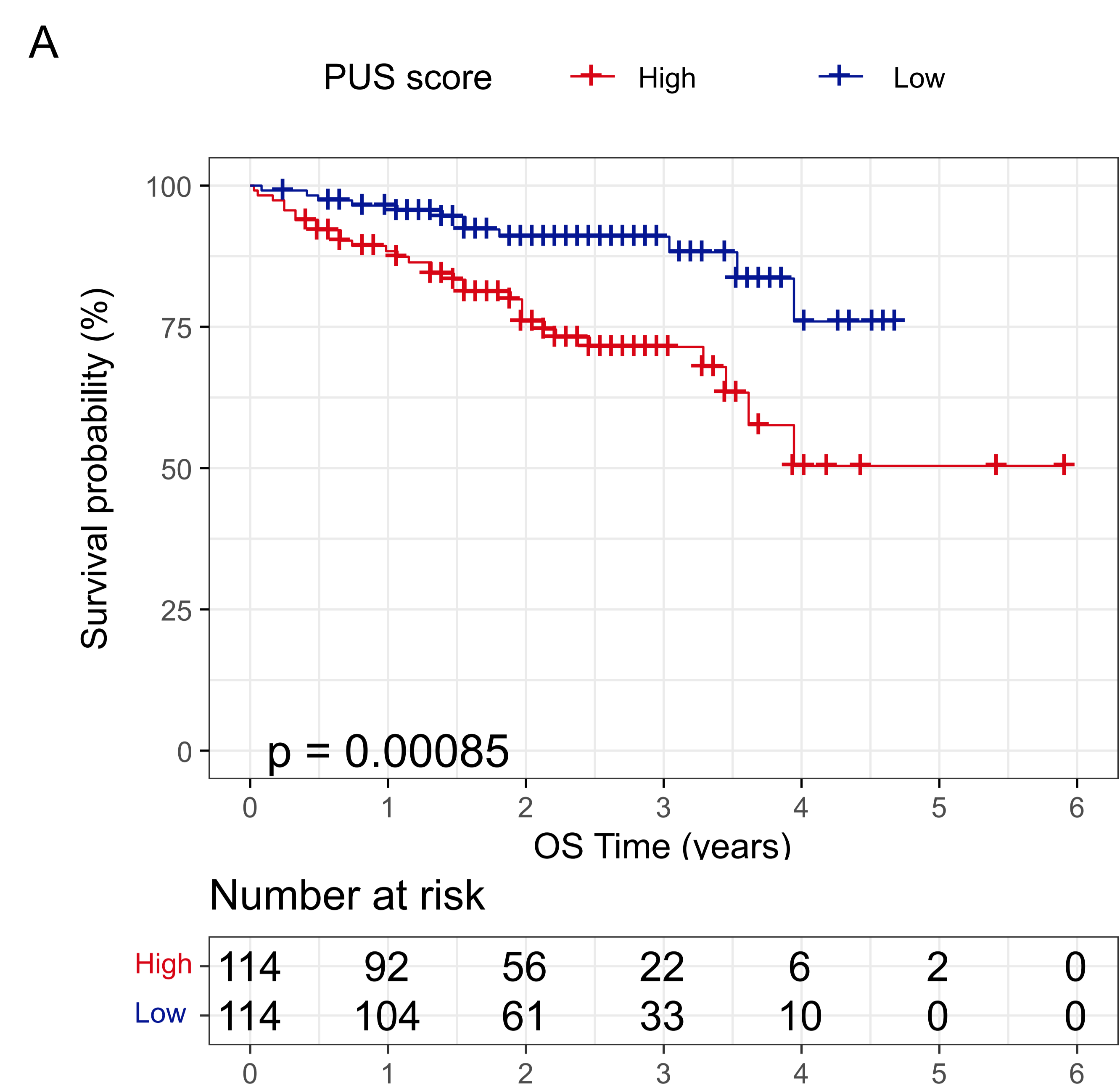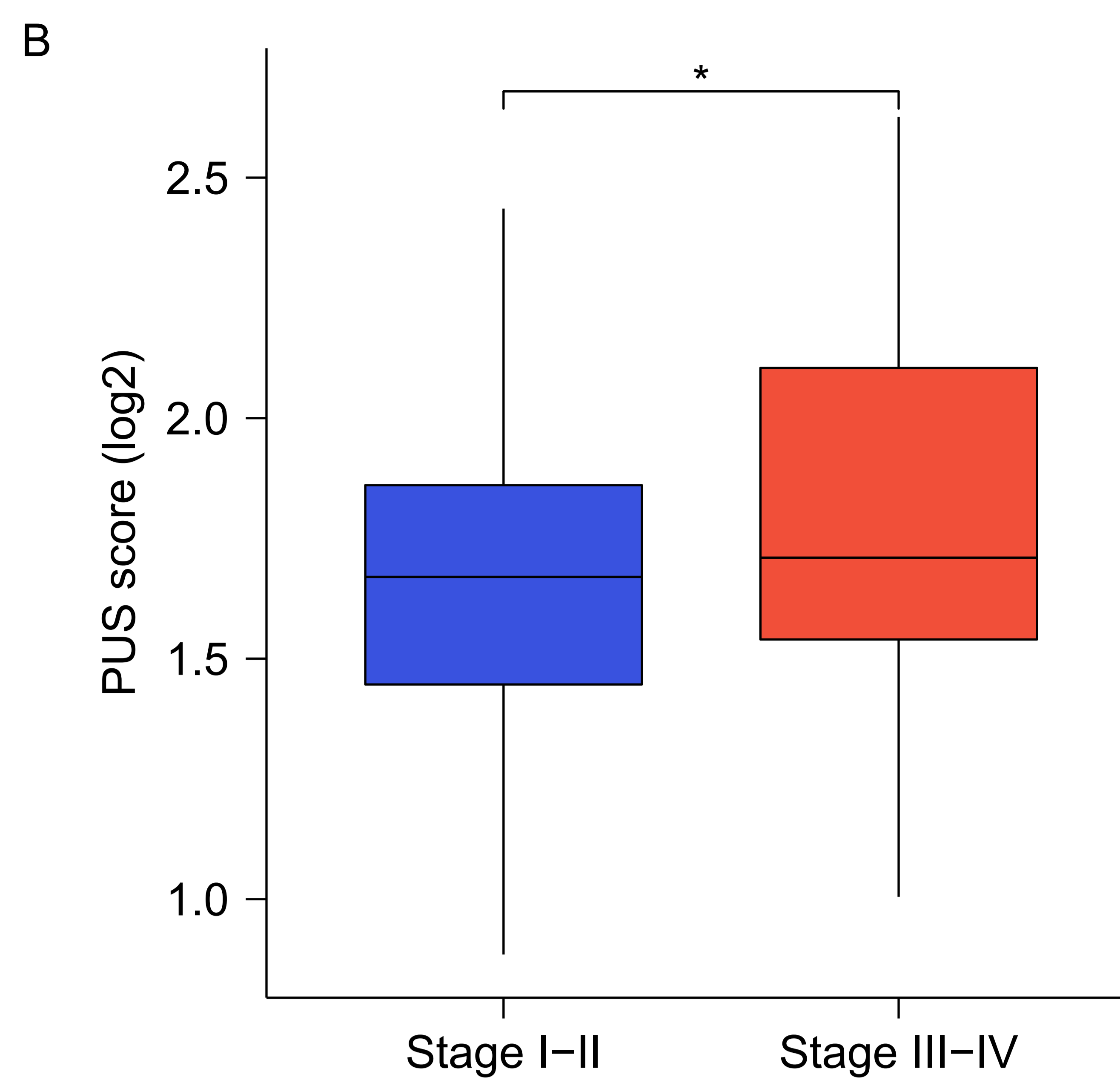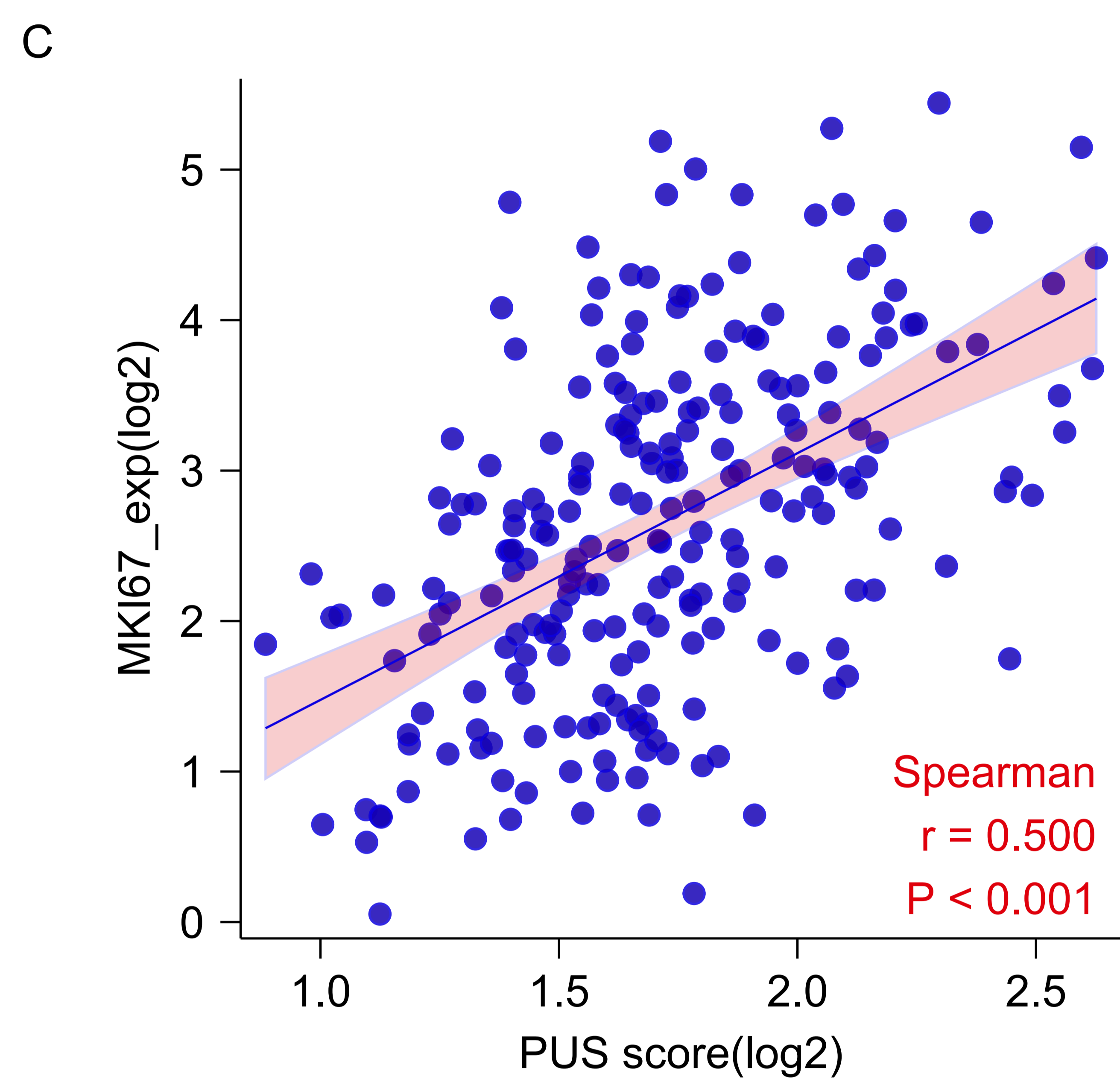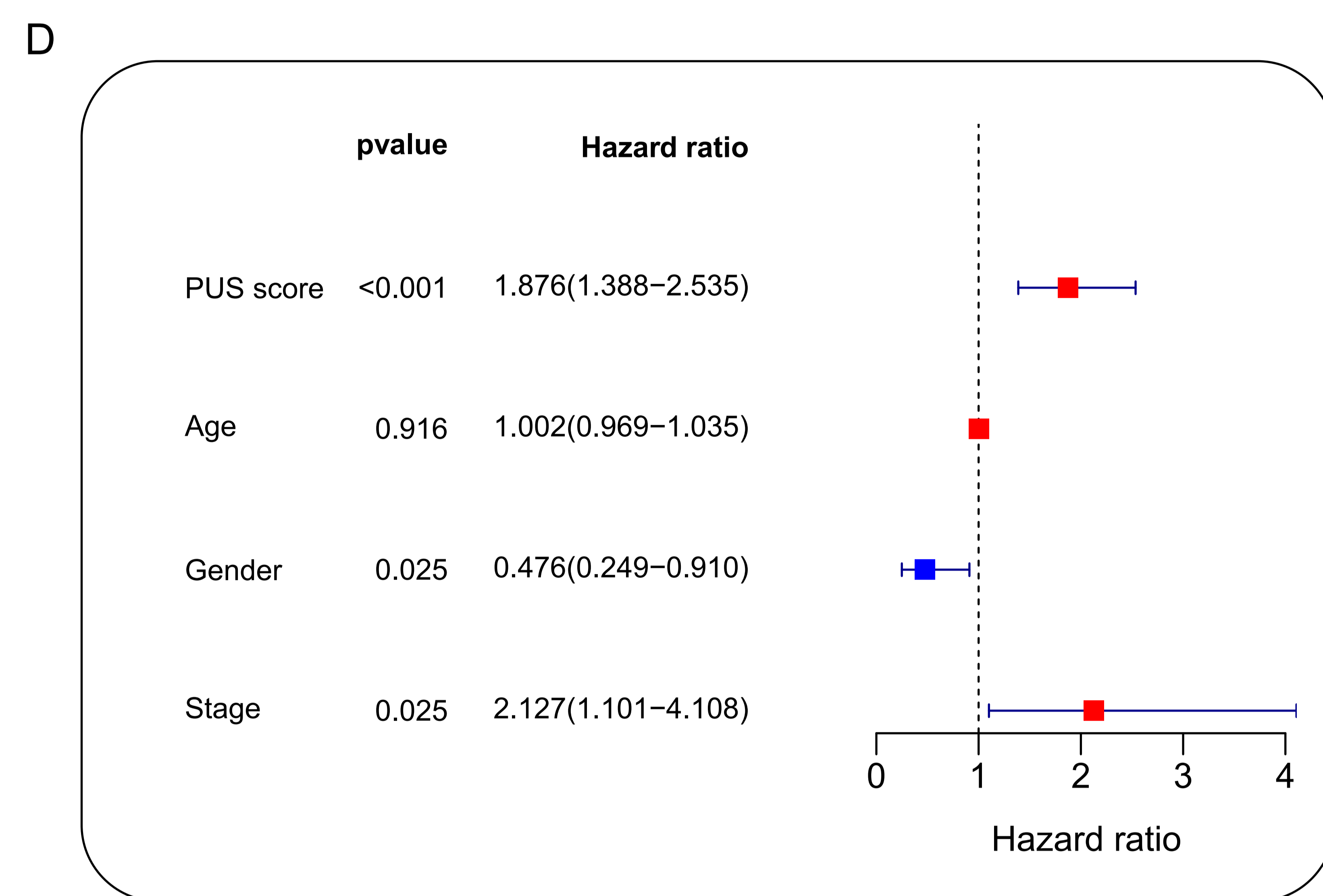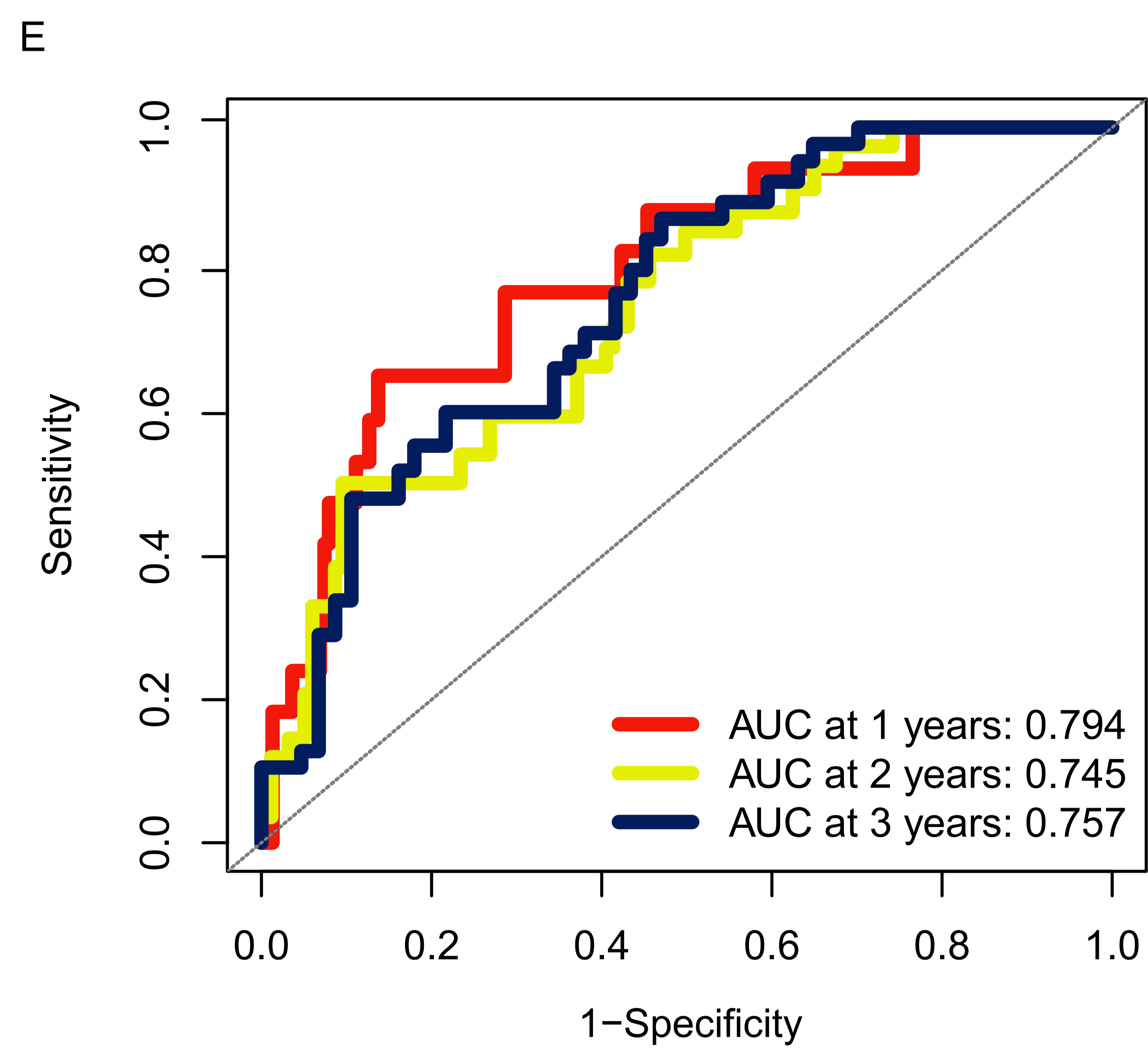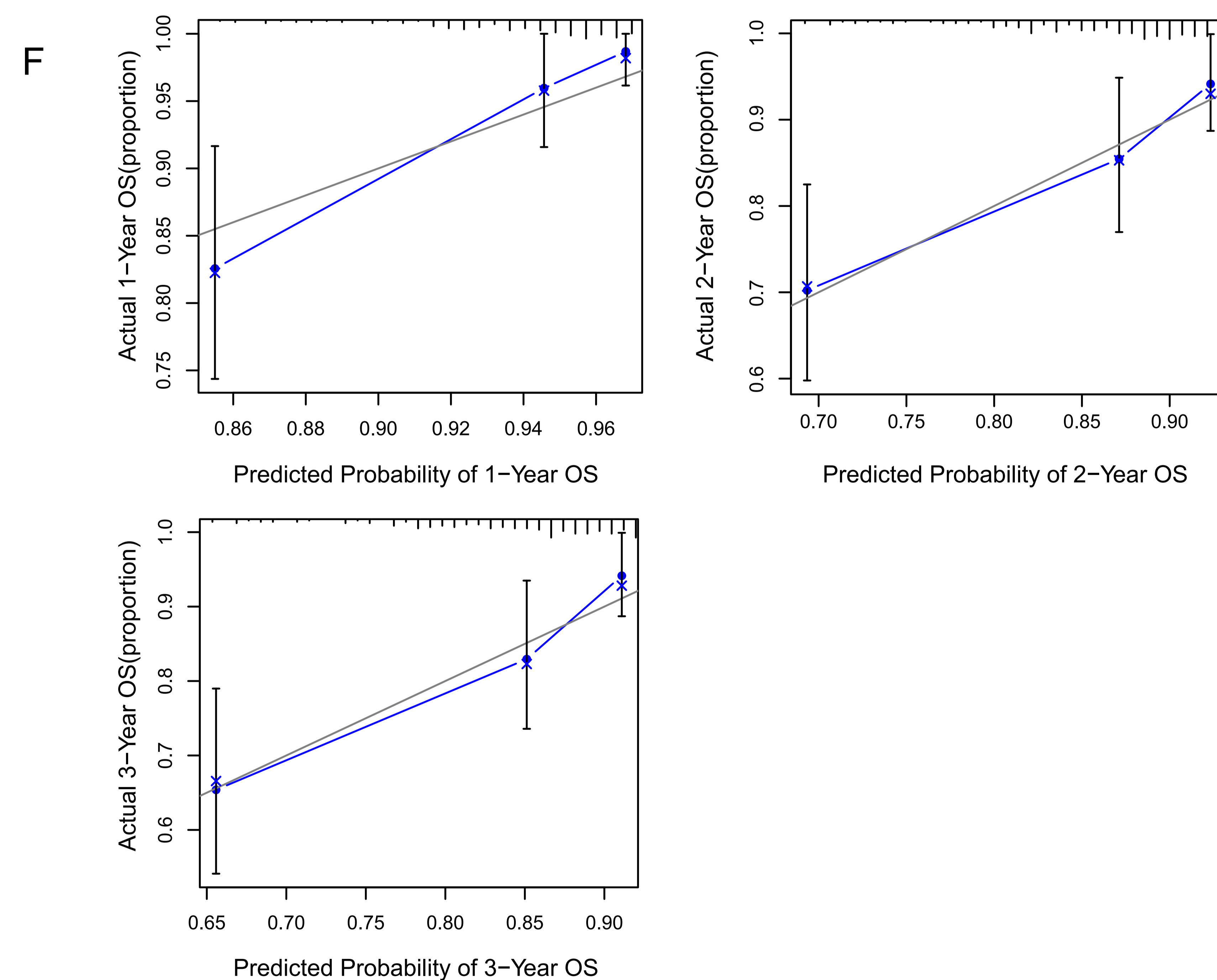

Supplement: Supplementary file 11 [file DataSheet5.PDF]
