# Supplementary material for: Integrative multiomics evaluation reveals the importance of pseudouridine synthases in hepatocellular carcinoma
Source: Front Genet. 2022 Nov 10;13:944681. doi: 10.3389/fgene.2022.944681 (PMC9686406; doi:10.3389/fgene.2022.944681)
Supplement: Supplementary file 5 [file DataSheet6.PDF]

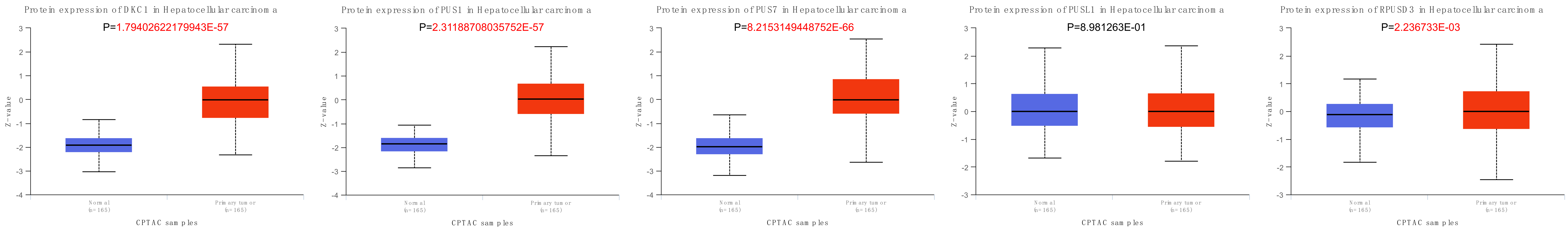
Protein expression of DKC1 in Hepatocellular carcinoma
P=1.79402622179943E-57
Z-value
3
2
1
0
-1
-2
-3
-4
Normal
(n=165)
Primary tumor
(n=165)
CPTAC samples
Protein expression of PUS1 in Hepatocellular carcinoma
P=2.31188708035752E-57
Z-value
Normal
(n=165)
Primary tumor
(n=165)
CPTAC samples
Protein expression of PUS7 in Hepatocellular carcinoma
P=8.2153149448752E-66
Z-value
Normal
(n=165)
Primary tumor
(n=165)
CPTAC samples
Protein expression of PUSL1 in Hepatocellular carcinoma
P=8.981263E-01
Z-value
Normal
(n=165)
Primary tumor
(n=165)
CPTAC samples
Protein expression of RPUSD3 in Hepatocellular carcinoma
P=2.236733E-03
Z-value
Normal
(n=165)
Primary tumor
(n=165)
CPTAC samples
